# Phenotypic age acceleration and omega-6/omega-3 PUFA ratio in dynamic atrial fibrillation–heart failure transitions: a multistate analysis

**DOI:** 10.1016/j.jnha.2026.100774

**Published:** 2026-01-12

**Authors:** Xianlin Zhang, Wenbo Tang, Pinfang Kang, Bi Tang, Zhongyan Du, Wenke Cheng

**Affiliations:** a Department of Cardiology, The First Affiliated Hospital of Bengbu Medical University, Bengbu, 233000, China; b The Second Clinical College of Anhui Medical University, 230000, Heifei, China; c Zhejiang Key Laboratory of Blood-Stasis-Toxin Syndrome, Zhejiang Chinese Medical University, Hangzhou, 310053, China; d School of Basic Medical Sciences, Zhejiang Chinese Medical University, Hangzhou, 310053, China; e Zhejiang Engineering Research Center for “Preventive Treatment” Smart Health of Traditional Chinese Medicine, Zhejiang Chinese Medical University, Hangzhou, 310053, China

**Keywords:** Phenotypic age acceleration, Omega-6, Omega-3, Atrial fibrillation, Heart failure, Cohort study

## Abstract

**Background:**

Biological aging and dietary fatty acid balance may influence the bidirectional progression between atrial fibrillation (AF) and heart failure (HF); however, most studies focus on single endpoints, overlooking intermediate states.

**Objective:**

To evaluate the independent and joint associations of phenotypic age acceleration (PhenoAgeAccel) and the plasma omega-6/omega-3 (ω-6/ω-3) polyunsaturated fatty acid (PFUA) ratio with AF-HF transitions, and to examine mediation by lipids and C-reactive protein.

**Methods:**

In a retrospective cohort of 191,091 UK Biobank participants free of baseline cardiovascular disease, PhenoAgeAccel was calculated as the residual from regressing phenotypic age on chronological age. The ω-6/ω-3 ratio was quantified by nuclear magnetic resonance. Incident AF and HF were modeled using clock-forward multistate Markov models for four transitions: baseline to AF, baseline to HF, AF to HF, and HF to AF. These transitions represent sequential disease progression, where either AF or HF may occur first and later progress to AF–HF comorbidity. Hazard ratios (HRs) were estimated per 1-SD increment. Joint exposure and mediation analyses were performed.

**Results:**

Over a median 15.4 years, 10,084 developed AF and 3,117 H F; 1,335 transitioned from AF to HF and 426 from HF to AF. Per 1-SD higher PhenoAgeAccel, risks increased for baseline-to-AF (HR 1.12 [95% CI 1.10–1.15]), baseline-to-HF (1.24 [1.21–1.26]), AF-to-HF (1.12 [1.09–1.15]), and HF-to-AF (1.06 [1.01–1.12]). Per 1-SD higher ω-6/ω-3 ratio, risks rose for baseline-to-AF (1.04 [1.02–1.06]), baseline-to-HF (1.07 [1.05–1.10]), AF-to-HF (1.12 [1.07–1.18]), and HF-to-AF (1.10 [1.01–1.20]). Mediation occurred via triglycerides (up to 38.5% of ω-6/ω-3–AF association) and CRP (up to 10.7% of PhenoAgeAccel–HF association).

**Conclusion:**

Higher PhenoAgeAccel and ω-6/ω-3 PFUA ratios were independently associated with higher risks of AF–HF transitions, with these associations partly explained by lipid and inflammatory pathways.

## Highlights

• PhenoAgeAccel and higher ω-6/ω-3 PUFA ratios independently increase risks of AF–HF dynamic transitions.
• PhenoAgeAccel and elevated ω-6/ω-3 ratios show joint associations with baseline-to-HF transition.
• Triglycerides and CRP partially mediate AF/HF transitions, implicating metabolic and inflammatory pathways.
• AF-to-comorbidity occurs earlier, while HF-to-comorbidity develops later.

## Graphical abstract

**Figure:**
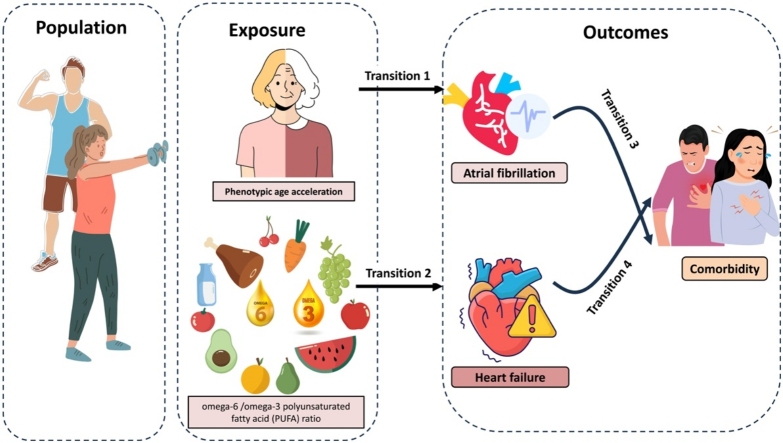

## 1 Introduction

Cardiovascular diseases (CVDs) remain the leading cause of global mortality, and their burden rises sharply with population aging [1,2]. Chronological age alone provides limited insight into risk heterogeneity, whereas biological aging reflects cumulative molecular, cellular, and physiological deterioration driven by inflammation, oxidative stress, and metabolic dysregulation [3]. Phenotypic Age (PhenoAge), developed by Levine et al., integrates nine routinely measured clinical biomarkers with chronological age to quantify systemic aging and has demonstrated superior predictive performance for mortality, cardiovascular events, and multimorbidity across diverse populations [3,4]. Phenotypic age acceleration (PhenoAgeAccel), defined as the residual of PhenoAge regressed on chronological age, captures whether individuals are biologically older or younger than expected and has been consistently associated with hypertension, diabetes, vascular dysfunction, and adverse cardiovascular outcomes [3,4].

Dietary factors also modulate aging–cardiovascular pathways through inflammatory and metabolic mechanisms. The plasma omega-6/omega-3 (ω-6/ω-3) polyunsaturated fatty acid (PUFA) ratio is a key modifiable marker that reflects the balance between pro-inflammatory ω-6 and anti-inflammatory ω-3 fatty acids [5]. Contemporary dietary patterns, characterized by high ω-6 intake and insufficient ω-3 sources, often produce ratios far above levels considered cardioprotective, and higher ω-6/ω-3 ratios have been linked to CVDs and arrhythmias in observational studies [6,7]. Yet, whether an elevated ω-6/ω-3 ratio interacts with accelerated biological aging to influence cardiovascular disease trajectories remains uncertain.

Atrial fibrillation (AF) and heart failure (HF) exemplify interconnected cardiovascular syndromes with substantial clinical and socioeconomic impact [[8], [9], [10]]. They frequently coexist and can precipitate each other through structural remodeling, hemodynamic compromise, and neurohormonal activation. Although prior studies have shown strong bidirectional risks, most have treated AF and HF as isolated endpoints and relied on standard Cox models that do not characterize intermediate states, recurrent transitions, or competing risks [[11], [12], [13]]. As a result, the dynamic evolution from a disease-free state to AF, HF, and their coexistence—and how this evolution may differ by biological aging or fatty acid balance—remains poorly understood.

To address these gaps, we applied multistate Markov models in the UK Biobank to evaluate how PhenoAgeAccel and the ω-6/ω-3 PUFA ratio influence four AF–HF transition pathways, thereby refining risk stratification beyond single-endpoint analyses.

## 2 Methods

### 2.1 Study design and population

This retrospective cohort analysis was conducted within the UK Biobank, a large prospective population-based study that recruited 502,128 participants aged 40–69 years between 2006 and 2010 across 22 assessment centres in the UK. At baseline, sociodemographic characteristics, lifestyle factors, physical measurements, and biological samples were collected via touchscreen questionnaires, interviews, and standardized examinations. Health outcomes were ascertained through linkage to national death registries and hospital episode statistics, and the detailed study design has been reported in detail elsewhere [14].

For the present sub-cohort analysis, we excluded participants with pre-existing cardiovascular diseases at baseline, including coronary artery disease (n = 27,620), heart failure (n = 881), valvular heart disease (n = 2,535), cardiomyopathy (n = 380), arrhythmias (n = 10,642), or stroke (n = 5801). Detailed diagnostic criteria are provided in Table S1. We further excluded those without ω-6/ω-3 PUFA ratio data (n = 206,878) or complete clinical biomarkers required for Phenotypic Age estimation (n = 36,559), those with current pregnancy (n = 64), and those with prevalent cancer (n = 19,676). The final analytic sample comprised 191,091 participants free of cardiovascular disease, cancer, and pregnancy at baseline.

The UK Biobank received ethical approval from the North West Multi-Centre Research Ethics Committee (ref. 21/NW/0157), and all participants provided written informed consent. Detailed information about the UK Biobank is available at http://www.ukbiobank.ac.uk/resources/. This study followed the STROBE guidelines for reporting observational studies.

### 2.2 Measurement of ω-6 and ω-3 polyunsaturated fatty acids

The primary exposure in this study was the plasma ω-6/ω-3 PUFA ratio, derived from UK Biobank Data-Field 23459. This ratio was computed by the UK Biobank as the absolute concentration (mmol/L) of total ω-6 PUFAs divided by that of total ω-3 PUFAs. Both were quantified in baseline non-fasting EDTA plasma samples using high-throughput proton nuclear magnetic resonance (NMR) spectroscopy on the Nightingale Health platform. NMR metabolomics data were sourced from the combined Phase 1 and Phase 2 releases, encompassing approximately 275,000 participants with available plasma ω-6 and ω-3 PUFA concentrations.

Blood samples were collected during baseline assessments (2006–2010), stored at −80 °C, and analyzed between 2019 and 2022 under standardized protocols for sample preparation, spectral acquisition, and quality control. These procedures included duplicate measurements, internal controls, dilution corrections, and cross-validation with clinical chemistry assays [[14], [15], [16]]. The NMR method yields highly reproducible results (intra-assay coefficients of variation <5%) and is well-suited for large-scale epidemiological analyses.

Additional details on ω-6 and ω-3 PUFA measurements in the UK Biobank are available at https://biobank.ndph.ox.ac.uk/showcase/refer.cgi?id=130

In line with evidence that ω-6/ω-3 ratios exceeding ∼10:1 are prevalent in Western diets and may elevate cardiovascular risks, we categorized participants into low (<10:1) and high (≥10:1) groups [17].

### 2.3 Biological aging assessment

Biological aging was assessed using the PhenoAge algorithm, a validated indicator of physiological dysregulation and mortality risk. Developed in the National Health and Nutrition Examination Survey (NHANES) III cohort, PhenoAge combines nine standard clinical biomarkers—albumin, creatinine, glucose, C-reactive protein, alkaline phosphatase, lymphocyte percentage, white blood cell count, mean cell volume, and red cell distribution width—with chronological age to compute a composite biological age in years [3]. The formula is as follows:

$$\text{PhenoAge}=141.50225+\frac{\text{ln}\left(-1.51714\times e^{xb}\right)}{0.09165}$$

$$x_{b}=-19.907-0.0336\times \text{Albumin}+0.0095\times \text{Creatinine}+0.1953\times \text{Glucose}+0.0954\times \text{ln}\left(\text{CRP}\right)-0.012\times \text{Lymphocyte}+0.0268\times \text{MCV}+0.3306\times \text{RDW}+0.00188\times \text{Alkaline Phosphatase}+0.0554\times \text{WBC}+0.0804\times \text{Chronological Age}$$

Phenotypic age acceleration (PhenoAgeAccel) was calculated as the residual from a linear regression of PhenoAge on chronological age. Positive residuals denote accelerated biological aging (biologically older than expected), while negative or near-zero residuals indicate decelerated or normative aging. Accordingly, PhenoAgeAccel was dichotomized into **Acceleration** (>0) and **Deceleration/Stasis** (≤0).

### 2.4 Ascertainment of HF and AF

Incident atrial fibrillation (AF) and heart failure (HF) were ascertained using the UK Biobank First Occurrences dataset (Category 1712), which aggregates data from primary care records, hospital inpatient admissions, death registries, and self-reports, mapped to 3-character I*nternational Classification of Diseases, 10th Revision* (ICD-10) codes. AF was defined as the first occurrence of ICD-10 code I48, and HF as I50. In this study, comorbidity is used to denote the coexistence of AF and HF. Participants were followed from baseline assessment (2006–2010) until the first occurrence of AF or HF, death, loss to follow-up, or administrative censoring. Outcome data for AF and HF were available through July 6, 2024, with mortality data (used for censoring in the multi-state model) updated to July 8, 2024; for consistency, July 6, 2024 was set as the observation end date. This ascertainment and follow-up strategy aligns with current UK Biobank data release protocols and established practices in cardiovascular research using this resource [18,19].

The primary outcome was the development of comorbidity between AF and HF. Disease progression was evaluated through four transition pathways: (1) baseline to incident HF, (2) baseline to incident AF, (3) HF to comorbidity, and (4) AF to comorbidity, as illustrated in Figure S1. These transitions reflect the natural clinical course in which individuals may develop AF or HF first and subsequently progress to the coexistence of both conditions, acknowledging that AF and HF often arise sequentially rather than as isolated, independent events.

### 2.5 Assessment of covariates

Baseline characteristics were ascertained from standardized touchscreen questionnaires, nurse-led interviews, physical measurements, and biochemical assays. Demographic factors included age, sex, and self-reported race (White vs. non-White). Socioeconomic status was evaluated using education level (high school or below vs. college or above), average household income (<£30,999 vs. ≥£30,999), and the Townsend Deprivation Index (TDI)—a validated area-based measure incorporating unemployment, car ownership, home ownership, and household overcrowding, with higher scores indicating greater deprivation.

Lifestyle factors encompassed physical activity, quantified via the International Physical Activity Questionnaire (IPAQ) and classified as low, moderate, or high; smoking status (current vs. non-current); alcohol consumption (current vs. non-current); and a cumulative dietary risk score based on nine food items (processed meat, red meat, total fish, milk, spreads, cereal, table salt, water, and fruit/vegetables) aligned with UK/European guidelines, and details are provided in Table S3. Each unhealthy item scored 1 point, yielding a total from 0 (healthiest) to 9 (unhealthiest), dichotomized as low risk (≤6 points) or high risk (≥7 points). Clinical factors included body mass index (BMI), hypertension, diabetes, and medication use (antihypertensives, lipid-lowering agents, and insulin); specific diagnostic criteria for hypertension and diabetes are detailed in Table S1.

To identify confounders in the associations between PhenoAgeAccel, the ω-6/ω-3 PUFA ratio, and AF/HF, we constructed a directed acyclic graph (DAG) using Dagitty (www.dagitty.net), as recommended in contemporary epidemiology for enhancing confounder selection and causal inference [20,21]. Socioeconomic indicators (education, income, TDI) were considered upstream factors mediated by proximal variables (e.g., diet patterns, physical activity) and thus not directly adjusted to prevent redundancy. Similarly, lipid profile and CRP were viewed as downstream mediators on the causal pathway—via metabolic and inflammatory mechanisms—and excluded to avoid overadjustment bias and ensure accurate estimation of the total effect [22].

The minimal sufficient adjustment set comprised age, BMI, sex, race, physical activity, hypertension, diabetes, dietary risk score, smoking status, alcohol consumption, antihypertensives, lipid-lowering agents, and insulin (Figure S2). These variables were selected as potential confounders or upstream determinants with plausible links to both exposure and outcomes, while avoiding those on the causal pathway. Furthermore, covariates were retained if they met at least one criterion for any outcome (AF or HF): (1) a >10% change in the β coefficient for the association between PhenoAgeAccel or the ω-6/ω-3 PUFA ratio and the outcome, or (2) a significant association with the outcome (P < 0.1) [23,24]. All qualifying covariates were included in the final models to ensure consistency and minimize residual confounding (Table S2).

### 2.6 Statistical analysis

Continuous variables were summarized as means ± standard deviations (SD), and categorical variables as frequencies (percentages). Associations between PhenoAgeAccel, the ω-6/ω-3 PUFA ratio, and disease transitions were evaluated using Cox proportional hazards models. Proportional hazards assumptions were verified via Schoenfeld residuals, with no substantial violations observed (Figure S3-S8). To model dynamic disease trajectories, we fitted clock-forward Markov multistate models using the mstate package in R, accounting for competing risks and temporal dependencies across four predefined transitions: baseline to HF, baseline to AF, HF to comorbidity, and AF to comorbidity (Figure S1). Hazard ratios (HRs) with 95% confidence intervals (CIs) were estimated per 1-SD increment in PhenoAgeAccel and the ω-6/ω-3 ratio. Participants (n = 757) developing comorbidity on the same day were excluded from multistate analyses due to indeterminate temporal ordering.

Exposure–response relationships were modeled using two complementary spline methods. In the overall cohort, Cox proportional hazards models incorporated restricted cubic splines (RCS) for PhenoAgeAccel and the ω-6/ω-3 PUFA ratio, evaluating 3–6 knots and selecting the optimal configuration via minimum Akaike Information Criterion. In the multistate framework, penalized splines (degrees of freedom = 3) were applied, offering enhanced stability for sparse samples or events by penalizing curve complexity, reducing knot placement sensitivity, and dampening the influence of extremes and local fluctuations [25]. As a sensitivity analysis, multistate RCS were fitted to confirm trend robustness. In both approaches, exposures were winsorized to the 1st–99th percentiles to minimize outlier effect.

Time-varying associations for transitions from HF or AF to comorbidity were assessed using piecewise Cox proportional hazards models. These included interaction terms between the exposures (per 1-SD increment in PhenoAgeAccel or the ω-6/ω-3 PUFA ratio) and predefined follow-up intervals (0–<1, 1–<3, 3–<5, and ≥5 years). Follow-up time was segmented with the survSplit function, and interval-specific HRs with 95%CIs were estimated and visualized in forest plots.

A joint exposure variable was created by cross-classifying PhenoAgeAccel (accelerated vs. decelerated/normative) and ω-6/ω-3 ratio (low vs. high), yielding four categories. The reference was decelerated/normative PhenoAgeAccel with low ratio (optimal profile); relative risks for HF and AF were compared across groups. Additive interaction was assessed via relative excess risk due to interaction (RERI), attributable proportion (AP), and synergy index (S), with 95% CIs from the delta method; multiplicative interaction was tested by including a product term and likelihood ratio comparison. Synergy was deemed present if both scales were significant.

Mediation by lipid profile and CRP was quantified using natural direct effects (NDE), natural indirect effects (NIE), total effects (TE), and proportion mediated (PM) for PhenoAgeAccel and ω-6/ω-3 ratio on HF, AF, and comorbidity, via the regmedint package with 1,000 bootstrap replications for 95% CIs.

Effect modification was further explored in subgroups by age (≤55 vs. >55 years), sex, ethnicity (White vs. non-White), dietary risk score (low vs. high), diabetes (yes/no), hypertension (yes/no), and physical activity (low, moderate, high). Interactions were tested via likelihood ratio comparisons of models with/without product terms; stratum-specific HRs (95% CIs) were reported per 1-SD increment.

Sensitivity analyses were conducted as follows: (1) participants with incident AF or HF within two years of enrollment were excluded to mitigate reverse causation; (2) missing covariates were multiply imputed and results were pooled using Rubin’s rules; (3) exposures were restricted to the 2.5th–97.5th percentiles; (4) complete-case analyses were performed after excluding participants with missing baseline data; (5) additional adjustment for lipid profile and CRP was undertaken in fully adjusted models; (6) a baseline-to-comorbidity transition was added to the multistate framework; (7) inverse probability weighting based on propensity scores was applied with PhenoAgeAccel and the ω-6/ω-3 ratio categorized according to prespecified rules, and covariate balance before and after weighting was evaluated using standardized mean differences (|SMD| < 0.10 indicating adequate balance); and (8) E-values were computed for statistically significant transitions involving PhenoAgeAccel or the ω-6/ω-3 ratio to quantify the minimum association strength, on the risk-ratio scale, that an unmeasured confounder would require with both exposure and outcome to fully explain the observed association conditional on measured covariates. All analyses were conducted in R version 4.2.3; two-sided tests were used, and P < 0.05 was considered statistically significant.

## 3 Results

A total of 191,091 participants were included, with a median follow-up of 15.38 years (interquartile range, 14.59–16.09). The overall cohort had a mean age of 55.79 ± 8.07 years, with 45.9% males. Sample sizes across the four HF/AF comorbidity transition pathways were baseline to AF (n = 10,084), baseline to HF (n = 3,117), AF to comorbidity (n = 1,335), and HF to comorbidity (n = 426). Absolute event rates per 1,000 person-years were 3.66 for baseline to AF, 1.12 for baseline to HF, 16.17 for AF to comorbidity, and 14.88 for HF to comorbidity. Temporal ordering was indeterminable for 757 participants because HF and AF occurred on the same day. Compared with the overall cohort, participants in all pathways were older, with higher proportions of males. They also exhibited higher prevalences of hypertension and diabetes; elevated body mass index, C-reactive protein, and triglyceride levels; and lower high-density lipoprotein levels. Use of antihypertensive and lipid-lowering medications was more common. Lifestyle factors included lower educational attainment and household income, higher cumulative dietary risk scores, increased current smoking rates, and greater proportions of non-drinkers. Detailed results are presented in Table 1.

**Table 1:** Baseline characteristics of the study population across multi-state transition groups.

Variable	Total	Baseline to AF	Baseline to HF	AF to Comorbidity	HF to Comorbidity
Sample size#	191091	10084	3117	1335	426
Age	55.79 (8.07)	61.19 (6.38)	60.81 (6.84)	62.21 (5.80)	61.80 (6.25)
TDI	−1.40 (3.04)	−1.35 (3.07)	−0.75 (3.29)	−1.03 (3.22)	−0.88 (3.30)
Men (%)	87747 (45.9%)	6608 (61.0%)	2322 (59.9%)	1317 (63.0%)	765 (64.7%)
White (**%**)	180420 (94.4%)	10553 (97.3%)	3653 (94.3%)	2024 (96.7%)	1130 (95.5%)
Hypertension (**%**)	46940 (24.6%)	4563 (42.1%)	1754 (45.3%)	1050 (50.2%)	591 (50.0%)
Diabetes (**%**)	8163 (4.3%)	857 (7.9%)	565 (14.6%)	261 (12.5%)	166 (14.0%)
Antihypertensives	32397 (17.0%)	3604 (33.3%)	1395 (36.0%)	893 (42.7%)	486 (41.2%)
Lowering lipids drugs	24645 (12.9%)	2492 (23.0%)	1061 (27.4%)	589 (28.2%)	346 (29.3%)
Insulin	1644 (0.9%)	160 (1.5%)	166 (4.3%)	51 (2.4%)	41 (3.5%)
BMI	27.33 (4.70)	28.74 (5.30)	29.51 (5.74)	29.94 (5.94)	30.15 (5.95)
CRP	2.52 (4.16)	3.09 (4.87)	3.87 (5.74)	3.77 (5.65)	3.86 (5.54)
HDL	1.46 (0.38)	1.41 (0.38)	1.36 (0.38)	1.39 (0.37)	1.36 (0.36)
LDL	3.61 (0.85)	3.51 (0.85)	3.54 (0.93)	3.43 (0.88)	3.45 (0.91)
TC	5.76 (1.11)	5.61 (1.12)	5.61 (1.21)	5.50 (1.16)	5.51 (1.19)
Triglycerides	1.74 (1.02)	1.80 (1.01)	1.95 (1.05)	1.85 (1.02)	1.90 (1.05)
Omega-6	4.54 (0.68)	4.45 (0.67)	4.47 (0.70)	4.41 (0.68)	4.42 (0.69)
Omega-3	0.53 (0.22)	0.53 (0.23)	0.52 (0.22)	0.51 (0.22)	0.51 (0.21)
Omega-6/ Omega-3	9.94 (4.37)	9.69 (4.21)	10.10 (4.72)	9.99 (4.30)	10.18 (5.35)
PhenoAge	53.43 (9.73)	60.20 (9.16)	61.79 (10.38)	62.66 (8.74)	62.90 (9.79)
PhenoAgeAccel	−2.36 (5.46)	−0.99 (6.56)	0.97 (8.20)	0.44 (6.79)	1.09 (7.86)
Phenotypic ageing					
Deceleration/Stasis	140798 (73.7%)	7034 (64.9%)	2090 (53.9%)	1144 (54.7%)	627 (53.0%)
Acceleration	50293 (26.3%)	3807 (35.1%)	1784 (46.1%)	948 (45.3%)	556 (47.0%)
Education					
University/College Degree or Equivalent	84862 (44.4%)	4396 (40.5%)	1381 (35.6%)	756 (36.1%)	428 (36.2%)
High-School Diploma or Below	104062 (54.5%)	6315 (58.3%)	2426 (62.6%)	1309 (62.6%)	736 (62.2%)
Unknown	2167 (1.1%)	130 (1.2%)	67 (1.7%)	27 (1.3%)	19 (1.6%)
Household_income,£					
< 30999	76391 (40.0%)	5333 (49.2%)	2129 (55.0%)	1161 (55.5%)	656 (55.5%)
≥30999	87611 (45.8%)	3733 (34.4%)	1002 (25.9%)	557 (26.6%)	313 (26.5%)
Unkown	27089 (14.2%)	1775 (16.4%)	743 (19.2%)	374 (17.9%)	214 (18.1%)
Cumulative dietary risk					
Low risk	149762 (78.4%)	8348 (77.0%)	2789 (72.0%)	1565 (74.8%)	866 (73.2%)
High risk	34446 (18.0%)	2031 (18.7%)	862 (22.3%)	415 (19.8%)	246 (20.8%)
Unknown	6883 (3.6%)	462 (4.3%)	223 (5.8%)	112 (5.4%)	71 (6.0%)
Physical activity					
Low	27114 (14.2%)	1533 (14.1%)	655 (16.9%)	323 (15.4%)	216 (18.3%)
Moderate	59830 (31.3%)	3186 (29.4%)	1076 (27.8%)	568 (27.2%)	311 (26.3%)
Hingh	61568 (32.2%)	3504 (32.3%)	1060 (27.4%)	617 (29.5%)	332 (28.1%)
Unkown	42579 (22.3%)	2618 (24.1%)	1083 (28.0%)	584 (27.9%)	324 (27.4%)
Smoking status					
Non-Active	170178 (89.1%)	9589 (88.5%)	3128 (80.7%)	1791 (85.6%)	979 (82.8%)
Active	20035 (10.5%)	1184 (10.9%)	718 (18.5%)	287 (13.7%)	194 (16.4%)
Unknown	878 (0.5%)	68 (0.6%)	28 (0.7%)	14 (0.7%)	10 (0.8%)
Drinking status					
Non-Active	14203 (7.4%)	805 (7.4%)	456 (11.8%)	186 (8.9%)	123 (10.4%)
Active	176439 (92.3%)	10006 (92.3%)	3399 (87.7%)	1898 (90.7%)	1052 (88.9%)
Unknown	449 (0.2%)	30 (0.3%)	19 (0.5%)	8 (0.4%)	8 (0.7%)

# indicates that HF and AF occurred on the same day in 757 participating groups, making it impossible to identify the disease temporal sequence.

BMI, body mass index; CRP, C-reactive protein; HDL, high-density lipoprotein; HF, heart failure; AF, atrial fibrillation; PUFA, polyunsaturated fatty acid.

## 4 Conventional cox models

In conventional Cox models, each 1-SD increment in PhenoAgeAccel was associated with increased risks of AF (HR, 1.14; 95% CI, 1.12–1.16), HF (HR, 1.20; 95% CI, 1.18–1.22), and AF/HF comorbidity (HR, 1.22; 95% CI, 1.19–1.25), as shown in the Table 2. When dichotomized, accelerated PhenoAge was linked to higher risks of AF (HR, 1.23; 95% CI, 1.18–1.28), HF (HR, 1.56; 95% CI, 1.47–1.65), and comorbidity (HR, 1.58; 95% CI, 1.45–1.72). For the ω-6/ω-3 PUFA ratio, each 1-SD increment was associated with elevated risks of AF (HR, 1.04; 95% CI, 1.03–1.06), HF (HR, 1.07; 95% CI, 1.05–1.09), and comorbidity (HR, 1.08; 95% CI, 1.06–1.10). When dichotomized, high ratio was associated with higher risks of AF (HR, 1.09; 95% CI, 1.05–1.13), HF (HR, 1.18; 95% CI, 1.12–1.25), and comorbidity (HR, 1.27; 95% CI, 1.17–1.38).

**Table 2:** Associations of PhenoAgeAccel and the ω-6/ω-3 PUFA ratio with dynamic transitions between atrial fibrillation and heart failure, evaluated using conventional Cox regression and multi-state models.

	Case	Proportion (%)	HR (95% CI)	*P*-value
**Traditional Cox model**				
PhenoAgeAccel				
*Per 1-SD increase*				
AF	11267	5.9%	1.14 (1.12, 1.16)	<0.001
HF	5209	2.7%	1.20 (1.18, 1.22)	<0.001
Comorbidity	2518	1.3%	1.22 (1.19, 1.25)	<0.001
*Acceleration verus Deceleration/Stasis*				
AF	11267	5.9%	1.23 (1.18, 1.28)	<0.001
HF	5209	2.7%	1.56 (1.47, 1.65)	<0.001
Comorbidity	2518	1.3%	1.58 (1.45, 1.72)	<0.001
**ω-6/ω-3 PUFA ratio**				
Per 1-SD increase				
AF	11267	5.9%	1.04 (1.03, 1.06)	<0.001
HF	5209	2.7%	1.07 (1.05, 1.09)	<0.001
Comorbidity	2518	1.3%	1.08 (1.06, 1.10)	<0.001
*Low versus high*				
AF	11267	5.9%	1.09 (1.05, 1.13)	<0.001
HF	5209	2.7%	1.18 (1.12, 1.25)	<0.001
Comorbidity	2518	1.3%	1.27 (1.17, 1.38)	<0.001
**Multi-state model**				
**PhenoAgeAccel**				
*Per 1-SD increase*				
Baseline → AF	10084	5.3%	1.12 (1.10, 1.15)	<0.001
Baseline → HF	3117	1.6%	1.24 (1.21, 1.26)	<0.001
AF → Comorbidity	1335	13.2%	1.12 (1.09, 1.15)	<0.001
HF → Comorbidity	426	13.7%	1.06 (1.01, 1.12)	0.02
*Acceleration verus Deceleration/Stasis*				
Baseline → AF	10084	5.3%	1.20 (1.15, 1.26)	0.001
Baseline → HF	3117	1.6%	1.67 (1.55, 1.81)	<0.001
AF → Comorbidity	1335	13.2%	1.38 (1.23, 1.55)	<0.001
HF → Comorbidity	426	13.7%	1.06 (0.86, 1.30)	0.588
**ω-6/ω-3 PUFA ratio**				
*Per 1-SD increase*				
Baseline → AF	10084	5.3%	1.04 (1.02, 1.06)	<0.001
Baseline → HF	3117	1.6%	1.07 (1.05, 1.10)	<0.001
AF → Comorbidity	1335	13.2%	1.12 (1.07, 1.18)	<0.001
HF → Comorbidity	426	13.7%	1.10 (1.01, 1.2)	0.022
*Low versus high*				
Baseline → AF	10084	5.3%	1.07 (1.03, 1.12)	0.001
Baseline → HF	3117	1.6%	1.18 (1.09, 1.27)	<0.001
AF → Comorbidity	1335	13.2%	1.23 (1.09, 1.37)	<0.001
HF → Comorbidity	426	13.7%	1.14 (0.93, 1.40)	0.199

PhenoAge, Phenotypic Age. ω-6/ω-3 PUFA ratio, ω-6: ω-3 polyunsaturated-fatty-acid ratio.

Traditional Cox models estimated the overall associations of PhenoAge acceleration and ω-6/ω-3 PUFA ratio with the incidence of atrial fibrillation, heart failure and comorbidity.

Multi-state models assessed hazard ratios for each transition pathway across disease progression stages.

All models were adjusted for age, body mass index, sex, race, physical activity, hypertension, diabetes, cumulative dietary-risk score, smoking status, alcohol consumption, use of antihypertensives, lipid-lowering agents, and insulin.

In multistate models, each 1-SD increment in PhenoAgeAccel was associated with increased risks across all transitions: baseline to AF (HR, 1.12; 95% CI, 1.10–1.15), baseline to HF (HR, 1.24; 95% CI, 1.21–1.26), AF to comorbidity (HR, 1.12; 95% CI, 1.09–1.15), and HF to comorbidity (HR, 1.06; 95% CI, 1.01–1.12), as shown in Table 2. When dichotomized, accelerated PhenoAge was linked to higher risks for baseline to AF (HR, 1.20; 95% CI, 1.15–1.26), baseline to HF (HR, 1.67; 95% CI, 1.55–1.81), and AF to comorbidity (HR, 1.38; 95% CI, 1.23–1.55), but not HF to comorbidity (HR, 1.06; 95% CI, 0.86–1.30). For the ω-6/ω-3 PUFA ratio, each 1-SD increment was positively associated with baseline to AF (HR, 1.04; 95% CI, 1.02–1.06), baseline to HF (HR, 1.07; 95% CI, 1.05–1.10), AF to comorbidity (HR, 1.12; 95% CI, 1.07–1.18), and HF to comorbidity (HR, 1.10; 95% CI, 1.01–1.20). When dichotomized, high ratio was associated with higher risks for baseline to AF (HR, 1.07; 95% CI, 1.03–1.12), baseline to HF (HR, 1.18; 95% CI, 1.09–1.27), and AF to comorbidity (HR, 1.23; 95% CI, 1.09–1.37), but not HF to comorbidity (HR, 1.14; 95% CI, 0.93–1.40).

## 5 Joint effects of phenotypic age acceleration and ω-6/ω-3 PUFA ratio on AF–HF transitions

Using decelerated/normative PhenoAgeAccel and low ω-6/ω-3 PUFA ratio as reference, joint effects were evident in conventional Cox models for AF, HF, and comorbidity, as shown in Table 3. For AF, elevated risks were observed with high ratio plus decelerated/normative PhenoAgeAccel (HR, 1.07; 95% CI, 1.02–1.12), accelerated PhenoAgeAccel plus low ratio (HR, 1.22; 95% CI, 1.16–1.28), and high ratio plus accelerated PhenoAgeAccel (HR, 1.33; 95% CI, 1.25–1.41). Similar patterns emerged for HF and comorbidity (all P < 0.001).

**Table 3:** Joint associations of phenotypic age acceleration and the ω-6/ω-3 PUFA ratio with AF, HF, and AF–HF comorbidit in conventional Cox and multistate models.

Phenotypic ageing	Deceleration/Stasis			Acceleration			
ω-6/ω-3 PUFA ratio	Low exposure	High exposure		Low exposure		High exposure	
Traditional Cox model		HR (95% CI)	*P* value	HR (95% CI)	*P* value	HR (95% CI)	*P* value
AF	1 (ref.)	1.07 (1.02,1.12)	*0.007*	1.22 (1.16, 1.28)	<0.001	1.33 (1.25, 1.41)	<0.001
HF	1 (ref.)	1.14 (1.05, 1.23)	<0.001	1.54 (1.43, 1.66)	<0.001	1.83 (1.68, 1.98)	<0.001
Comorbidity	1 (ref.)	1.24 (1.11, 1.39)	<0.001	1.58 (1.42, 1.76)	<0.001	1.96 (1.71, 2.2)	<0.001
Multi-state model							
Baseline → AF	1 (ref.)	1.06 (1.00, 1.12)	0.032	1.20 (1.13, 1.26)	<0.001	1.27 (1.19, 1.35)	<0.001
Baseline → HF	1 (ref.)	1.09 (0.99, 1.21)	0.087	1.60 (1.45, 1.76)	<0.001	1.91 (1.72, 2.12)	<0.001
AF → Comorbidity	1 (ref.)	1.24 (1.06, 1.44)	0.007	1.39 (1.20, 1.62)	<0.001	1.64 (1.38, 1.94)	<0.001
HF → Comorbidity	1 (ref.)	1.18 (0.87, 1.59)	0.28	1.08 (0.81, 1.43)	0.604	1.18 (0.84, 1.59)	0.256

AF, atrial fibrillation; HF, heart failure; PUFA, polyunsaturated fatty acid; HR, hazard ratio; CI, confidence interval. Reference group: PhenoAgeAccel *Deceleration/Stasis* with Low ω-6/ω-3 ratio.

Models were adjusted for age, body mass index, sex, race, physical activity, hypertension, diabetes, cumulative dietary-risk score, smoking status, alcohol consumption, use of antihypertensives, lipid-lowering agents, and insulin.

In multistate models, joint effects were significant across most transitions. For baseline to AF, higher risks were noted with high ratio plus decelerated/normative PhenoAgeAccel (HR, 1.06; 95% CI, 1.00–1.12), accelerated PhenoAgeAccel plus low ratio (HR, 1.20; 95% CI, 1.13–1.26), and high ratio plus accelerated PhenoAgeAccel (HR, 1.27; 95% CI, 1.19–1.35). For baseline to HF, risks were elevated with accelerated PhenoAgeAccel plus low ratio (HR, 1.60; 95% CI, 1.45–1.76) and high ratio plus accelerated PhenoAgeAccel (HR, 1.91; 95% CI, 1.72–2.12), but not high ratio plus decelerated/normative PhenoAgeAccel (HR, 1.09; P = 0.087). For AF to comorbidity, all combinations were significant (all P ≤ 0.007). No significant associations were found for HF to comorbidity (all P > 0.25).

Most interactions were nonsignificant on additive and multiplicative scales (Table S4). However, positive additive interaction was detected for baseline to HF between high ω-6/ω-3 ratio and accelerated PhenoAgeAccel (RERI, 0.221 [95% CI, 0.047–0.396]; P = 0.013; AP, 0.116 [95% CI, 0.024–0.207]; P = 0.013; S, 1.32 [95% CI, 1.013–1.627]; P = 0.019), whereas multiplicative interaction was not (HR, 1.095 [95% CI, 0.947–1.265]; P = 0.219).

## 6 Temporal effects of the ω-6/ω-3 PUFA ratio and PhenoAgeAccel on HF/AF transitions

**Fig. 1:**
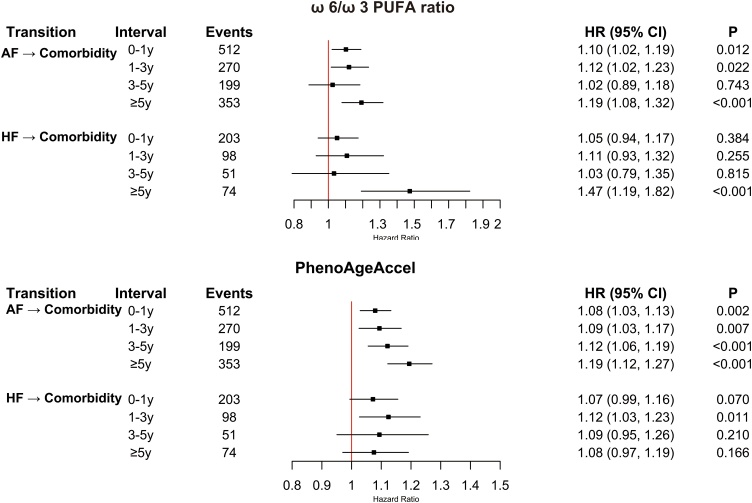
Time-stratified associations between biological PhenoAgeAccel, omega-6/omega-3 PUAF ratio and risk of comorbidity following AF or HF. PhenoAgeAccel, Phenotypic Age Acceleration; ω-6/ω-3 PUFA ratio, omega-6/omega-3 polyunsaturated fatty acid ratio. Hazard ratios and 95% confidence intervals were estimated for the associations between biological age acceleration (assessed using PhenoAgeAccel) and omega-6/omega-3 ratio with subsequent risk of comorbidity across time intervals after AF or HF (0–1 years, 1–3 years, 3–5 years, and ≥5 years). Models were adjusted for age, body mass index, sex, race, physical activity, hypertension, diabetes, cumulative dietary-risk score, smoking status, alcohol consumption, antihypertensive use, lipid-lowering medication use, and insulin use.

As shown in Fig. 1, for the AF-to-comorbidity transition, each 1-SD increment in the ω-6/ω-3 PUFA ratio was associated with elevated risk during 0–<1 year (HR, 1.10; 95% CI, 1.02–1.19), 1–<3 years (HR, 1.12; 95% CI, 1.02–1.23), and ≥5 years (HR, 1.19; 95% CI, 1.08–1.32), but not 3–<5 years (P = 0.743). Similarly, PhenoAgeAccel was linked to higher risk during 0–<1 year (HR, 1.08; 95% CI, 1.03–1.13), 1–<3 years (HR, 1.09; 95% CI, 1.03–1.17), 3–<5 years (HR, 1.12; 95% CI, 1.06–1.19), and ≥5 years (HR, 1.19; 95% CI, 1.12–1.27).

For the HF-to-comorbidity transition, the ω-6/ω-3 PUFA ratio showed association only during ≥5 years (HR, 1.47; 95% CI, 1.19–1.82; P < 0.001), with no associations during 0–<1, 1–<3, or 3–<5 years (all P > 0.1). For the HF-to-comorbidity transition, per 1-SD increase in PhenoAgeAccel, a borderline association was observed in the 0–<1-year interval (HR = 1.07; 95% CI, 0.99–1.16; P = 0.070) and a significant association in 1–<3 years (HR = 1.12; 95% CI, 1.03–1.23), with no associations in 3–<5 years (P = 0.210) or ≥5 years (P = 0.166).

## 7 Exposure–response across multistate HF/AF transitions

**Fig. 2:**
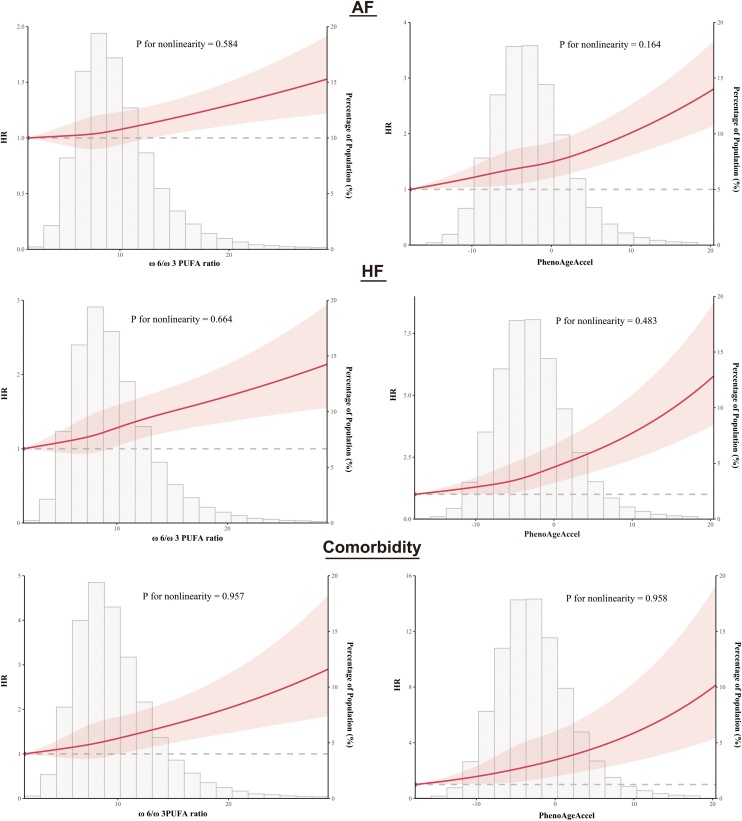
Restricted cubic spline analyses of associations between ω-6/ω-3 PUFA Ratio, PhenoAgeAccel and the incidence of atrial fibrillation, heart failure, and comorbidity. Models were adjusted for age, sex, race, body mass index, physical activity, hypertension, diabetes, cumulative dietary-risk score, smoking status, alcohol consumption, antihypertensive use, lipid-lowering medication use, and insulin use.

In the overall cohort, RCS analyses showed no significant nonlinearity between the ω-6/ω-3 PUFA ratio and AF, HF, or comorbidity (all P for nonlinearity >0.1; Fig. 2). Likewise, no nonlinearity was observed for PhenoAgeAccel with AF, HF or comorbidity (all P for nonlinearity >0.1; Fig. 2).

In multistate models, penalized splines demonstrated gradual risk increases with rising ω-6/ω-3 PUFA ratio and PhenoAgeAccel for incident AF, incident HF, and AF-to-comorbidity transitions (Figure S9). For HF-to-comorbidity, the trend was less robust, with wider confidence intervals indicating greater uncertainty. These patterns were consistent with multistate RCS analyses (Figure S10).

## 8 Mediating roles of lipid profile and CRP in associations between PhenoAgeAccel and ω-6/ω-3 ratio with HF/AF transitions

For PhenoAgeAccel with AF, positive mediation occurred via LDL (PM, 4.71%) and TC (PM, 3.70%), whereas HDL showed competitive mediation (PM, −1.24%). For the ω-6/ω-3 PUFA ratio with AF, mediation was strongest via TG (PM, 38.5%), TC (PM, 26.86%), and LDL (PM, 25.05%); CRP mediated modestly (PM, 2.98%), with HDL competitive (PM, −8.53%). For PhenoAgeAccel with HF, CRP mediated positively (PM, 10.72%), while HDL was competitive (PM, −0.35%). For the ω-6/ω-3 ratio with HF, CRP mediated modestly (PM, 5.47%). For PhenoAgeAccel with AF/HF comorbidity, CRP mediated strongly (PM, 9.36%), followed by LDL (PM, 3.01%) and TC (PM, 2.07%); HDL was competitive (PM, −1.05%). For the ω-6/ω-3 ratio with comorbidity, mediation was strongest via LDL and TC (each PM, 13.97%), with modest CRP mediation (PM, 4.93%). Detailed results are provided in Figures S11 and S12.

## 9 Subgroup and sensitivity analyses

Subgroup analyses demonstrated limited effect modification. Significant interactions were identified only for race (P for interaction = 0.009), dietary risk score (P for interaction = 0.024), and diabetes (P for interaction = 0.006) in the AF-to-comorbidity transition, whereas no interactions were observed for other characteristics or for the ω-6/ω-3 ratio (all P for interaction >0.05; Figure S13).

Sensitivity analyses addressing reverse causation, outlier influence, missing data, model specification, and covariate balance, including IPW, yielded results consistent with the primary analyses, supporting the robustness of our conclusions. Full results are presented in Tables S5–S15.

## 10 Discussion

This UK Biobank study demonstrates that higher PhenoAgeAccel and an elevated ω-6/ω-3 PUFA ratios are independently associated with increased risks of dynamic transitions between AF and HF, including from baseline to AF or HF and subsequent bidirectional progressions to comorbidity. Joint analyses indicated additive interaction, particularly for the baseline-to-HF transition, while temporal patterns suggested sustained associations over long-term follow-up. Mediation by lipid profiles and CRP was observed, especially for ω-6/ω-3 PUFA ratio through triglycerides and low-density lipoprotein. Effect modification for PhenoAgeAccel was noted in the AF-to-HF transition, with stronger links in White participants, those with higher dietary risk scores, and individuals without diabetes. Collectively, these results incorporate biological aging and dietary fatty acid imbalance into a multistate framework, offering insights into AF-HF trajectories that extend beyond single-endpoint models.

Our findings align with and build on existing evidence that PhenoAgeAccel predicts cardiovascular outcomes and mortality more effectively than chronological age. Developed by Levine et al., PhenoAge-based metrics have shown superior prognostic value for all-cause mortality, CVD incidence, and multimorbidity in diverse cohorts [3]. Data from the NHANES associate accelerated phenotypic age with higher risks of hypertension-related mortality and diabetic complications [26,27]. Similarly, elevated ω-6/ω-3 PUFA ratio have been linked to adverse cardiovascular and mortality outcomes in prospective studies, though discussions continue regarding whether the ratio itself is more important than the absolute levels of ω-3 [7,28]. Meta-analyses suggest that ω-3 supplementation may lower CVD mortality by approximately 10% in certain populations [29]. The bidirectional AF-HF relationship is established, with AF potentially leading to HF through hemodynamic and rate-related effects, and HF facilitating AF via structural and electrical remodeling; in the Framingham Heart Study, incident AF roughly doubled HF risk [30], and recent reviews highlight high comorbidity rates and poorer prognosis [9]. Multistate models improve upon standard Cox regression by explicitly modeling transitions, competing risks, and time dependencies, as seen in studies of AF-HF biomarker associations and post-myocardial infarction pathways [11,31]. By applying this approach to PhenoAgeAccel and the PUFA ratio, our analysis reveals transition-specific patterns and their interactions. Importantly, PhenoAge was originally developed and calibrated in the NHANES cohort. When the same algorithm was applied in the UK Biobank, the parameter structure of its nine biomarkers remained highly consistent with the original model, indicating good reproducibility within this large European cohort [32]. Moreover, several UK Biobank–based studies have reported that PhenoAge and PhenoAgeAccel retain strong predictive performance for mortality, cardiovascular disease, cancer, and multimorbidity [4,[33], [34], [35]]. These findings support the external applicability of PhenoAge within the UK Biobank population and provide a solid rationale for its use in the present analysis.

These associations are biologically plausible, potentially involving inflammation, oxidative stress, endothelial dysfunction, and cardiac remodeling. An elevated ω-6/ω-3 PUFA ratio promotes pro-inflammatory eicosanoids from arachidonic acid, increasing cytokines such as interleukin-6 and tumor necrosis factor-alpha, which contribute to oxidative damage and endothelial impairment [6]. In contrast, ω-3-derived resolvins and protectins exhibit anti-inflammatory properties, supporting nitric oxide availability and possibly slowing atherosclerosis [36]. PhenoAgeAccel, which includes indicators like CRP and glucose, reflects systemic dysregulation that may exacerbate atrial fibrosis and ventricular changes, enabling AF to induce cardiomyopathy and HF to promote atrial dilation with neurohormonal involvement [37]. Consistent with this inflammation-lipid pathway, our mediation results showed indirect effects through triglycerides (up to 38.5% for AF) and CRP (up to 10.7% for HF). However, because CRP is one of the nine biomarkers included in the PhenoAge algorithm, partial dependence between the exposure and mediator may modestly inflate the indirect effects, and mediation proportions involving CRP should therefore be interpreted with caution. Temporal findings—stronger early links for AF-to-HF and more delayed signals for HF-to-AF—align with acute inflammatory responses versus gradual structural alterations, supported by experimental data where ω-3 reduces oxidative stress in HF models [38]. Although the hazard ratios for the HF-to-AF comorbidity transition were modest (approximately 1.06–1.10), this pattern is clinically coherent. Individuals with established HF already have a substantial intrinsic propensity to develop AF due to chronically elevated filling pressures, atrial stretch, progressive fibrosis, and neurohormonal activation. Because the underlying risk of developing AF is already very high in patients with HF, any additional increase in risk will look relatively small in terms of the hazard ratio, even though the absolute increase in risk may still be clinically meaningful. The limited number of HF-to-AF events in our cohort likely attenuated effect estimates through regression-dilution and sparse-data bias. Importantly, in a population with such high underlying risk, even modest relative increases that persist over long-term follow-up may still translate into clinically relevant absolute risk. Moreover, stronger effects among participants without diabetes may reflect diabetes-specific pathways (e.g., hyperglycemia-induced stress) overshadowing aging effects, while those with poor diets could involve synergistic inflammation from nutrient deficiencies; racial variations (stronger in Whites) may stem from genetic differences in PUFA metabolism but could also arise from low non-White representation and reduced statistical power in minorities [[39], [40], [41]].

From a clinical perspective, these findings may support the potential integration of PhenoAgeAccel and the ω-6/ω-3 PUFA ratio into cardiovascular risk assessment. PhenoAgeAccel can be readily implemented because it is derived from nine routine laboratory biomarkers, allowing automated calculation without additional testing burden. Although PUFA profiling currently requires targeted lipid assays, our linear dose–response results indicate that progressively lower ω-6/ω-3 ratios are consistently associated with reduced risks of AF, HF, and AF–HF comorbidity, reinforcing dietary recommendations favoring increased intake of fish, nuts, seeds, and EPA/DHA supplements, as supported by selected trials reporting reductions in AF recurrence or HF events [42,43]. The temporal findings, with the strongest effects emerging after at least five years of follow-up, underscore the importance of early identification and sustained lifestyle modification. Moreover, the joint-exposure analyses showed that individuals with both accelerated PhenoAgeAccel and a high ω-6/ω-3 ratio experienced substantially higher risks of first AF and HF (27% and 91% increases, respectively), highlighting a vulnerable subgroup that may benefit from intensified preventive strategies. Although direct interventional evidence remains limited, existing data suggest that improving metabolic health and reducing dietary ω-6/ω-3 imbalance could potentially contribute to attenuate long-term progression toward AF–HF multimorbidity.

Strengths of this study include the large, detailed UK Biobank cohort with extended follow-up; validated biomarker-derived aging assessments; reliable metabolomics for PUFA measurement; and a multistate model capturing clinical disease progression, including less common transitions. Confounding was minimized through directed acyclic graph-based adjustments and IPW, with mediation and interaction analyses adding mechanistic context. Sensitivity analyses supported robustness: IPW balanced covariates well, yielding estimates similar to the primary models; E-values (1.24–2.73) suggest that only moderately strong unmeasured confounding could nullify these associations.

Limitations include the observational design, which prevents causal conclusions, and potential residual confounding from factors like genetics or evolving behaviors. First, residual confounding cannot be completely ruled out. Although we used DAG-based confounder selection and IPW to reduce bias, unmeasured or incompletely measured factors such as genetic susceptibility (including APOE genotype), broader dietary patterns, and physical fitness may still influence both the exposures and AF–HF transitions. In addition, despite excluding individuals with baseline cardiovascular disease and conducting sensitivity analyses that removed incident events within the first two years of follow-up to mitigate reverse causation, preclinical or subclinical cardiac dysfunction present at baseline may still have contributed to early disease processes that influence these associations. These limitations suggest that our estimates may remain partly affected by unmeasured confounding or early underlying pathology. Second, both PhenoAgeAccel and the ω-6/ω-3 PUFA ratio were measured only at baseline. Temporal changes in biological aging and fatty acid profiles, whether driven by lifestyle modification, age-related metabolic shifts, or the development of comorbidities, could not be captured. This within-person variability typically leads to regression-dilution bias and tends to bias effect estimates toward the null, implying that the true associations may be stronger than observed. Future cohort studies with repeated biomarker measurements are needed to clarify how longitudinal trajectories of biological aging and PUFA balance relate to AF–HF transitions over time. The UK Biobank's selection bias toward healthier, mostly White volunteers may reduce generalizability. Additionally, NMR-measured PUFAs reflect circulating rather than tissue concentrations.

## 11 Conclusions

In summary, PhenoAgeAccel and the ω-6/ω-3 PUFA ratio were associated with higher risks across AF-HF transitions, with evidence of additive interaction and subgroup differences that identify vulnerable populations. These observations may help inform strategies that integrate aging biomarkers with dietary modifications in the context of CVD prevention.

## CRediT authorship contribution statement

Conceptualization: XLZ and WKC; Methodology: WKC; Formal analysis and investigation: ZXL, WBT, nd WKC iting—original draft preparation: XLZ, WBT and WKC; Writing—review and editing: XLZ, WBT, PFK, BT, ZYD, and WKC; Resources: WKC; Supervision: ZYD and WKC. All authors contributed to subsequent revisions and approved the final version. All authors read and approved the final manuscript.

## Consent for publication

Not applicable.

## Ethics approval and consent to participate

The UK Biobank was established with ethical clearance from the North West Multi-Centre Research Ethics Committee (REC reference: 11/NW/0382). Written informed consent have been provided by all participants.

## Funding

This work was supported by grants from the Natural Science Research Project of Anhui Educational Committee (No. 2022AH051462) and Research Project of Zhejiang Chinese Medical University (2023JKZKTS11).

## Data availability

Data can be accessed from a public and open repository. Interested researchers can apply for access to the UK Biobank data at https://www.ukbiobank.ac.uk and https://biobank.ndph.ox.ac.uk/ukb.

## Declaration of competing interest

The authors declare that they have no conflict of interest.
