# Supplementary material for: Phenotypic age acceleration and omega-6/omega-3 PUFA ratio in dynamic atrial fibrillation–heart failure transitions: a multistate analysis
Source: J Nutr Health Aging. 2026 Jan 12;30(2):100774. doi: 10.1016/j.jnha.2026.100774 (PMC12901530; doi:10.1016/j.jnha.2026.100774)

Supplement

Phenotypic Age Acceleration, Omega-6/Omega-3 Polyunsaturated Fatty Acid Ratio, and Dynamic AF–HF Transitions: A Multistate Analysis

Xianlin Zhang , Wenbo Tang , Pinfang Kang , Bi Tang , Zhongyan Du, Wenke Cheng

Table S1. Diagnostic codes for diseases

Diseases	ICD-9 diagnosis	ICD-10 diagnosis	Self-report [a]	Primary Care [b]	Medication [c] / Operation code, self-reported [d]
Hypertension	401, 4010, 4011, 4019, 402, 4020, 4021, 4029, 403, 4030, 4031, 4039, 404, 4040, 4041, 4049, 405, 4050, 4051, 4059	I10-I13, I15	20002	Category 3000	Blood pressure medication / NA
Diabetes	2500, 25000, 25001, 25009, 2501, 25011, 25019, 2503, 2504, 2505, 25099	E10-E14	20002	Category 3000	Insulin/ NA
CAD	410, 4109, 411, 4119, 412, 4129, 413, 4139, 4140, 4148, 4149	I20-I25	20002	Category 3000	NA / Coronary angioplasty (PTCA) +/- stent; Coronary artery bypass grafts (CABG); Triple heart bypass
Stroke	430-434, 436	I60-64,Field 40001, Field 40002	20002	Category 3000	NA
Heart failure	428, 4280, 4281, 4289	I50	20002	Category 3000	NA
cardiomyopathy	422, 4229, 425, 4251, 4254	I40-I43	20002	Category 3000	NA
Heart valve disease	394, 3940, 3942, 3949, 395, 3951, 3959, 396, 3969, 424, 4240, 4241, 4243, 4249	I05-I09, I34-I38	20002	Category 3000	NA / Aortic valve repair/replacement; Mitral valve repair/replacement; Other valve repair/replacement
Arrhythmia	426, 4260, 4261, 4263, 4264, 4265, 4266, 4267, 4269, 427, 4270, 4271, 4273, 4274, 4276, 4278, 4279	I44, I45, I47-I49	20002	Category 3000	NA / Pacemaker; Defibrillator insertion; Cardiac ablation

ICD: International Classification of Disease. NA, not applicable.

[a] ICD-10 codes in the Hospital inpatient data and Death Register records

[b] 20002 is the data code used in UK Biobank: Non-cancer illness code, self-reported.

[c] This category contains data on primary care data recorded by health professionals working at general practices.

[d] Data-field 6153 and 6177.

[e] Data-Field 20004

Table S2. Covariate selection based on change-in-estimate and statistical significance criteria.

Variable	Beta change percent (%)	P value	Include		Beta change percent (%)	P value	Include
**PhenoAgeAccel-AF**				**ω-6/ω-3 PUFA ratio-AF**			
Age	0.84	< 0.001	Yes	Age	220	< 0.001	Yes
BMI	20.1	< 0.001	Yes	BMI	7.08	< 0.001	Yes
Sex	8.38	< 0.001	Yes	Sex	51	< 0.001	Yes
Race	3.04	< 0.001	Yes	Race	5.5	< 0.001	Yes
Physical activity	0.34	< 0.001	Yes	Physical activity	1.43	< 0.001	Yes
Hypertension	15.2	< 0.001	Yes	Hypertension	69.8	< 0.001	Yes
Diabetes	11.4	< 0.001	Yes	Diabetes	14.3	< 0.001	Yes
Diet score	0.4	< 0.001	Yes	Diet score	13	< 0.001	Yes
Smoking status	0.14	< 0.001	Yes	Smoking status	7.42	< 0.001	Yes
Alcohol consumption	0.14	0.275	No	Alcohol consumption	1.93	0.275	No
Antihypertensives	16.7	< 0.001	Yes	Antihypertensives	80	< 0.001	Yes
Lipid-lowering agents	11.8	< 0.001	Yes	Lipid-lowering agents	95.8	< 0.001	Yes
Insulin	1.72	< 0.001	Yes	Insulin	1.1	< 0.001	Yes
**PhenoAgeAccel-HF**				**ω-6/ω-3 PUFA ratio-HF**			
Age	2.87	< 0.001	Yes	Age	251	< 0.001	Yes
BMI	14.6	< 0.001	Yes	BMI	30.1	< 0.001	Yes
Sex	2.38	< 0.001	Yes	Sex	84.9	< 0.001	Yes
Race	0.75	0.145	No	Race	2.96	0.145	No
Physical activity	0.5	< 0.001	Yes	Physical activity	3.64	< 0.001	Yes
Hypertension	8.78	< 0.001	Yes	Hypertension	118	< 0.001	Yes
Diabetes	14.6	< 0.001	Yes	Diabetes	53.5	< 0.001	Yes
Diet score	0.33	< 0.001	Yes	Diet score	62.8	< 0.001	Yes
Smoking status	0.67	< 0.001	Yes	Smoking status	76.8	< 0.001	Yes
Alcohol consumption	2.63	< 0.001	Yes	Alcohol consumption	37	< 0.001	Yes
Antihypertensives	7.4	< 0.001	Yes	Antihypertensives	132	< 0.001	Yes
Lipid-lowering agents	8.68	< 0.001	Yes	Lipid-lowering agents	198	< 0.001	Yes
Insulin	7.15	< 0.001	Yes	Insulin	6.4	< 0.001	Yes

PhenoAge, Phenotypic Age. ω-6/ω-3 PUFA ratio, ω-6 : ω-3 polyunsaturated-fatty-acid ratio.
BMI, body mass index.

Table S3. The calculation method for cumulative dietary risk

Variable	Categories reported from the touch-screen questionnaire	Binary Variables
Fruit & vegetables (regrouped from fruit, dried fruit & Vegetable)	Serving/day	≥5 serving/day (Ref.)<br><5 serving/day
Total fish intake (regrouped from Both total non-oily fish and oily fish)	Never<br>Less than once a week<br>Once a week<br>2-4 times a week<br>5-6 times a week<br>Once or more daily	≥2 times a week (at least once a week of each category)<br>(Ref.)<br>< once a week of each one
Processed meat intake	Never<br>Less than once a week<br>Once a week<br>2-4 times a week<br>5-6 times a week<br>Once or more daily	≤Once a week<br>(Ref.)<br>> Once a week
Red meat (regrouped from beef, pork and lamb)	Never<br>Less than once a week<br>Once a week<br>2-4 times a week<br>5-6 times a week<br>Once or more daily	≤Once a week (Ref.)<br>>Once a week
Milk type used	Full cream<br>Semi-skimmed<br>Skimmed<br>Soya<br>another type of milk<br>Never rarely have milk	Semi-skimmed/skimmed (Ref.)<br>Full cream/ another type of milk/ never rarely have milk
Spread type	Never/rarely<br>Butter<br>Other type/ margarine<br>Flora pro-active/benecol	Never/rarely (Ref.)<br>Another selection
Cereal intake	Bowls/week	>5 bowls (Ref)<br>≤5 bowls
Salt added to food	Never/rarely<br>Sometimes<br>Usually<br>Always	Never/rarely (Ref.)<br>Another selection
Water intake	Glasses/day	≥6 glasses (Ref.)<br><6 glasses

Table S4. Additive and Multiplicative Interactions between ω-6/ω-3 Polyunsaturated Fatty Acid Ratio and Phenotypic Aging on Risks of Incident AF, HF, Comorbidity, and Their Dynamic Transitions

	**Additive interactive**					**Multiplicative interactive HR (95%CI)**	**P-value**
	**Measure**	**Estimate**	**Lower**	**Upper**			
AF	RERI	0.038	-0.057	0.133	0.43		
	AP	0.029	-0.042	1.0	0.425	1.02 (0.94, 1.10)	0.665
	S	1.13	0.771	1.496	<0.001		
HF	RERI	0.125	-0.034	0.284	0.123		
	AP	0.07	-0.017	0.157	0.115	1.03 (0.92, 1.15)	0.604
	S	1.189	0.917	1.459	<0.001		
	RERI	0.118	-0.125	0.361	0.339		
Comorbidity	AP	0.061	-0.062	0.184	0.329	0.99 (0.843, 1.16)	0.898
	S	1.455	0.818	1.473	<0.001		
Baseline → AF	RERI	0.018	-0.082	0.117	0.726		
	AP	0.014	-0.064	0.092	0.724	1.005 (0.923, 1.094)	0.909
	S	1.07	0.661	1.479	<0.001		
Baseline → HF	RERI	0.221	0.047	0.396	**0.013**		
	AP	0.116	0.024	0.207	**0.013**	1.095 (0.947, 1.265)	0.219
	S	1.32	1.013	1.627	**0.019**		
	RERI	0.007	-0.299	0.313	0.964		
AF → Comorbidity	AP	0.004	-0.182	0.191	0.964	0.95 (0.761, 1.187)	0.652
	S	1.011	0.522	1.501	<0.001		
	RERI	-0.071	-0.516	0.375	0.755		
HF → Comorbidity	AP	-0.06	-0.438	0.319	0.757	0.993 (0.631, 1.381)	0.73
	S	0.723	-0.631	2.077	0.295		

AF, atrial fibrillation. HF, heart failure. RERI, relative excess risk due to interaction; AP, attributable proportion due to interaction; S, synergy index; HR, hazard ratio; CI,confidence interval;

Additive interaction models were adjusted for age, body mass index, sex, race, physical activity, hypertension, diabetes, cumulative dietary-risk score, smoking status, alcohol consumption, use of antihypertensives, lipid-lowering agents, and insulin.

Multiplicative interaction models were adjusted for the same set of covariates as the main models, with an additional product term included to capture the interaction between the ω-6/ω-3 PUFA ratio and phenotypic aging status.

Table S5. Associations of PhenoAgeAccel and the ω-6/ω-3 PUFA Ratio With Dynamic AF–HF Transitions: Cox and Multistate Analyses Excluding Events in the First Two Years of follow-up.

	Case	Proportion (%)	HR (95% CI)	*P*-value
**Multi-state model**				
**PhenoAgeAccel**				
Per 1-SD increase				
Baseline → AF	9357	4.9%	1.12 (1.09, 1.14)	<0.001
Baseline → HF	2974	1.6%	1.24 (1.22, 1.26)	<0.001
AF → Comorbidity	1206	12.9%	1.16 (1.12, 1.20)	<0.001
HF → Comorbidity	393	13.2%	1.09 (1.03, 1.16)	0.006
Acceleration verus Deceleration/Stasis				
Baseline → AF	9357	4.9%	1.19 (1.13, 1.24)	0.001
Baseline → HF	2974	1.6%	1.67 (1.54, 1.80)	<0.001
AF → Comorbidity	1206	12.9%	1.43 (1.26, 1.61)	<0.001
HF → Comorbidity	393	13.2%	1.08 (0.87, 1.33)	0.493
**ω-6/ω-3 PUFA ratio**				
Per 1-SD increase				
Baseline → AF	9357	4.9%	1.04 (1.02, 1.06)	<0.001
Baseline → HF	2974	1.6%	1.07 (1.05, 1.09)	<0.001
AF → Comorbidity	1206	12.9%	1.10 (1.05, 1.16)	<0.001
HF → Comorbidity	393	13.2%	1.10 (1.01, 1.2)	0.021
Low versus high				
Baseline → AF	9357	4.9%	1.08 (1.03, 1.12)	0.001
Baseline → HF	2974	1.6%	1.16 (1.08, 1.25)	<0.001
AF → Comorbidity	1206	12.9%	1.18 (1.05, 1.33)	0.007
HF → Comorbidity	393	13.2%	1.18 (0.96, 1.46)	0.121

PhenoAge, Phenotypic Age. ω-6/ω-3 PUFA ratio, ω-6 : ω-3 polyunsaturated-fatty-acid ratio.
Multi-state models assessed hazard ratios for each transition pathway across disease progression stages.
Multi-state models were adjusted for age, body mass index, sex, race, physical activity, hypertension, diabetes, cumulative dietary-risk score, smoking status, alcohol consumption, use of antihypertensives, lipid-lowering agents, and insulin.

Table S6. Multistate associations of PhenoAgeAccel and the ω-6/ω-3 PUFA ratio with AF–HF transitions (exposures restricted to the central 95%).

	Case	Proportion (%)	HR (95% CI)	*P*-value
**Multi-state model**				
**PhenoAgeAccel**				
Per 1-SD increase				
Baseline → AF	9514	5.3%	1.09 (1.07, 1.12)	<0.001
Baseline → HF	2774	1.5%	1.28 (1.24, 1.33)	<0.001
AF → Comorbidity	1217	12.8%	1.16 (1.10, 1.23)	<0.001
HF → Comorbidity	364	13.1%	1.03 (0.93, 1.14)	0.58
Acceleration verus Deceleration/Stasis				
Baseline → AF	9514	5.3%	1.17 (1.12, 1.22)	<0.001
Baseline → HF	2774	1.5%	1.52 (1.40, 1.65)	<0.001
AF → Comorbidity	1217	12.8%	1.30 (1.15, 1.46)	<0.001
HF → Comorbidity	364	13.1%	0.97 (0.78, 1.21)	0.818
**ω-6/ω-3 PUFA ratio**				
Per 1-SD increase				
Baseline → AF	9551	5.3%	1.05 (1.03, 1.07)	<0.001
Baseline → HF	2942	1.6%	1.10 (1.06, 1.14)	<0.001
AF → Comorbidity	1257	13.2%	1.13 (1.06, 1.19)	<0.001
HF → Comorbidity	401	13.6%	1.03 (0.93, 1.14)	0.509
Low versus high				
Baseline → AF	9551	5.3%	1.07 (1.02, 1.12)	0.002
Baseline → HF	2942	1.6%	1.15 (1.06, 1.24)	<0.001
AF → Comorbidity	1257	13.2%	1.19 (1.06, 1.34)	0.004
HF → Comorbidity	401	13.6%	1.13 (0.92, 1.39)	0.258

PhenoAge, Phenotypic Age. ω-6/ω-3 PUFA ratio, ω-6 : ω-3 polyunsaturated-fatty-acid ratio.
Multi-state models assessed hazard ratios for each transition pathway across disease progression stages.
Multi-state models were adjusted for age, body mass index, sex, race, physical activity, hypertension, diabetes, cumulative dietary-risk score, smoking status, alcohol consumption, use of antihypertensives, lipid-lowering agents, and insulin.

Table S7. Associations of PhenoAgeAccel and the ω-6/ω-3 PUFA ratio with AF–HF transitions: multistate models pooled across five multiply imputed datasets (Rubin's rules).

	Case	Proportion (%)	HR (95% CI)	*P*-value
**Multi-state model**				
**PhenoAgeAccel**				
Per 1-SD increase				
Baseline → AF	10084	5.3%	1.13 (1.11, 1.15)	<0.001
Baseline → HF	3117	1.6%	1.23 (1.21, 1.25)	<0.001
AF → Comorbidity	1335	13.2%	1.11 (1.08, 1.15)	<0.001
HF → Comorbidity	426	13.7%	1.06 (1.01, 1.12)	0.02
Acceleration verus Deceleration/Stasis				
Baseline → AF	10084	5.3%	1.20 (1.15, 1.26)	0.001
Baseline → HF	3117	1.6%	1.64 (1.52, 1.77)	<0.001
AF → Comorbidity	1335	13.2%	1.37 (1.22, 1.54)	<0.001
HF → Comorbidity	426	13.7%	1.06 (0.86, 1.30)	0.59
**ω-6/ω-3 PUFA ratio**				
Per 1-SD increase				
Baseline → AF	10084	5.3%	1.04 (1.02, 1.06)	<0.001
Baseline → HF	3117	1.6%	1.07 (1.05, 1.10)	<0.001
AF → Comorbidity	1335	13.2%	1.12 (1.07, 1.18)	<0.001
HF → Comorbidity	426	13.7%	1.11 (1.02, 1.20)	0.018
Low versus high				
Baseline → AF	10084	5.3%	1.07 (1.03, 1.12)	<0.001
Baseline → HF	3117	1.6%	1.17 (1.09, 1.27)	<0.001
AF → Comorbidity	1335	13.2%	1.22 (1.09, 1.37)	<0.001
HF → Comorbidity	426	13.7%	1.16 (0.95, 1.42)	0.155

PhenoAge, Phenotypic Age. ω-6/ω-3 PUFA ratio, ω-6 : ω-3 polyunsaturated-fatty-acid ratio.

Multi-state models assessed hazard ratios for each transition pathway across disease progression stages.

Multi-state models were adjusted for age, body mass index, sex, race, physical activity, hypertension, diabetes, cumulative dietary-risk score, smoking status, alcohol consumption, use of antihypertensives, lipid-lowering agents, and insulin.

Table S8. Associations of PhenoAgeAccel and the ω-6/ω-3 PUFA Ratio With Dynamic AF–HF Transitions: Conventional Cox and Multistate Analyses in a Complete-Case Sample.

	Case	Proportion (%)	HR (95% CI)	*P*-value
**Multi-state model**				
**PhenoAgeAccel**				
Per 1-SD increase				
Baseline → AF	5809	4.6%	1.11 (1.08, 1.14)	<0.001
Baseline → HF	1570	1.2%	1.21 (1.17, 1.25)	<0.001
AF → Comorbidity	681	11.7%	1.16 (1.11, 1.22)	<0.001
HF → Comorbidity	202	12.9%	0.99 (0.91, 1.09)	0.907
Acceleration verus Deceleration/Stasis				
Baseline → AF	5809	4.6%	1.17 (1.10, 1.24)	0.001
Baseline → HF	1570	1.2%	1.51 (1.36, 1.68)	<0.001
AF → Comorbidity	681	11.7%	1.41 (1.20, 1.66)	<0.001
HF → Comorbidity	202	12.9%	0.99 (0.74, 1.34)	0.973
**ω-6/ω-3 PUFA ratio**				
Per 1-SD increase				
Baseline → AF	5809	4.6%	1.02 (0.99, 1.04)	0.146
Baseline → HF	1570	1.2%	1.07 (1.04, 1.10)	<0.001
AF → Comorbidity	681	11.7%	1.11 (1.06, 1.23)	<0.001
HF → Comorbidity	202	12.9%	1.12 (1.02, 1.23)	0.014
Low versus high				
Baseline → AF	5809	4.6%	1.04 (0.99, 1.10)	0.124
Baseline → HF	1570	1.2%	1.15 (1.04, 1.27)	0.009
AF → Comorbidity	681	11.7%	1.23 (1.05, 1.44)	0.009
HF → Comorbidity	202	12.9%	1.25 (0.94, 1.66)	0.119

PhenoAge, Phenotypic Age. ω-6/ω-3 PUFA ratio, ω-6 : ω-3 polyunsaturated-fatty-acid ratio.
Multi-state models assessed hazard ratios for each transition pathway across disease progression stages.
Multi-state models were adjusted for age, body mass index, sex, race, physical activity, hypertension, diabetes, cumulative dietary-risk score, smoking status, alcohol consumption, use of antihypertensives, lipid-lowering agents, and insulin.

Table S9. Multistate associations of PhenoAgeAccel and the ω-6/ω-3 PUFA ratio with transitions between atrial fibrillation and heart failure, with additional adjustment for CRP, HDL, triglycerides, total cholesterol, and LDL.

	Case	Proportion (%)	HR (95% CI)	*P*-value
**Multi-state model**				
**PhenoAgeAccel**				
Per 1-SD increase				
Baseline → AF	10084	5.3%	1.12 (1.10, 1.14)	<0.001
Baseline → HF	3117	1.6%	1.23 (1.21, 1.25)	<0.001
AF → Comorbidity	1335	13.2%	1.11 (1.08, 1.15)	<0.001
HF → Comorbidity	426	13.7%	1.06 (1.01, 1.12)	0.029
Acceleration verus Deceleration/Stasis				
Baseline → AF	10084	5.3%	1.17 (1.12, 1.23)	<0.001
Baseline → HF	3117	1.6%	1.60 (1.47, 1.72)	<0.001
AF → Comorbidity	1335	13.2%	1.32 (1.17, 1.49)	<0.001
HF → Comorbidity	426	13.7%	1.05 (0.85, 1.30)	0.653
**ω-6/ω-3 PUFA ratio**				
Per 1-SD increase				
Baseline → AF	10084	5.3%	1.02 (1.0, 1.04)	0.053
Baseline → HF	3117	1.6%	1.08 (1.05, 1.10)	<0.001
AF → Comorbidity	1335	13.2%	1.13 (1.07, 1.18)	<0.001
HF → Comorbidity	426	13.7%	1.10 (1.01, 1.2)	0.025
Low versus high				
Baseline → AF	10084	5.3%	1.03 (0.99, 1.08)	0.175
Baseline → HF	3117	1.6%	1.19 (1.10, 1.28)	<0.001
AF → Comorbidity	1335	13.2%	1.24 (1.10, 1.39)	<0.001
HF → Comorbidity	426	13.7%	1.15 (0.93, 1.41)	0.191

PhenoAge, Phenotypic Age. ω-6/ω-3 PUFA ratio, ω-6 : ω-3 polyunsaturated-fatty-acid ratio.
Traditional Cox models estimated the overall associations of PhenoAge acceleration and ω-6/ω-3 PUFA ratio with the incidence of atrial fibrillation ,heart failure and comorbidity.
Multi-state models assessed hazard ratios for each transition pathway across disease progression stages.
All models were adjusted for age, body mass index, sex, race, physical activity, hypertension, diabetes, cumulative dietary-risk score, smoking status, alcohol consumption, use of antihypertensives, lipid-lowering agents, insulin, CRP, HDL, TG, TC, and LDL.

Table S10. Associations of PhenoAgeAccel and the ω-6/ω-3 PUFA ratio with dynamic AF–HF transitions in multistate models, including a baseline-to-comorbidity transition.

	Case	Proportion (%)	HR (95% CI)	*P*-value
**Multi-state model**				
**PhenoAgeAccel**				
Per 1-SD increase				
Baseline → AF	10084	5.3%	1.12 (1.10, 1.14)	<0.001
Baseline → HF	3117	1.6%	1.24 (1.22, 1.27)	<0.001
Baseline → Comorbidity	757	0.4%	1.2 (1.14, 1.26)	<0.001
AF → Comorbidity	1335	13.2%	1.12 (1.09, 1.16)	<0.001
HF → Comorbidity	426	13.7%	1.06 (1.01, 1.1)	0.027
Acceleration verus Deceleration/Stasis				
Baseline → AF	10084	5.3%	1.20 (1.15, 1.25)	0.001
Baseline → HF	3117	1.6%	1.68 (1.56, 1.82)	<0.001
Baseline → Comorbidity	757	0.4	1.59 (1.36, 1.85)	<0.001
AF → Comorbidity	1335	13.2%	1.38 (1.23, 1.55)	<0.001
HF → Comorbidity	426	13.7%	1.06 (0.86, 1.30)	0.584
**ω-6/ω-3 PUFA ratio**				
Per 1-SD increase				
Baseline → AF	10084	5.3%	1.04 (1.02, 1.06)	<0.001
Baseline → HF	3117	1.6%	1.07 (1.05, 1.10)	<0.001
Baseline → Comorbidity	757	0.4%	1.07 (1.03, 1.12)	<0.001
AF → Comorbidity	1335	13.2%	1.12 (1.07, 1.18)	<0.001
HF → Comorbidity	426	13.7%	1.10 (1.01, 1.2)	0.02
Low versus high				
Baseline → AF	10084	5.3%	1.07 (1.03, 1.12)	0.002
Baseline → HF	3117	1.6%	1.18 (1.09, 1.27)	<0.001
Baseline → Comorbidity	757	0.4	1.29 (1.11, 1.50)	<0.001
AF → Comorbidity	1335	13.2%	1.22 (1.09, 1.37)	<0.001
HF → Comorbidity	426	13.7%	1.15 (0.94, 1.41)	0.174

PhenoAge, Phenotypic Age. ω-6/ω-3 PUFA ratio, ω-6 : ω-3 polyunsaturated-fatty-acid ratio.
Multi-state models assessed hazard ratios for each transition pathway across disease progression stages.
Multi-state models were adjusted for age, body mass index, sex, race, physical activity, hypertension, diabetes, cumulative dietary-risk score, smoking status, alcohol consumption, use of antihypertensives, lipid-lowering agents, and insulin.

Table S11. Covariate Balance Assessment Before and After Inverse Probability Weighting Based on PhenoAgeAccel Group Stratification (Acceleration verus Deceleration/Stasis).

Variables	level	Standardized Mean Difference (Unadjusted)	Type	Standardized Mean Difference (Adjusted or Weighted)
Age	NA	0.003804	Continuous	0.018581
Sex (men)	0	0.239024	Categorical	0.005641
Sex (women)	1	0.239024	Categorical	0.005641
Race (White)	0	0.062122	Categorical	0.005349
Race (others)	1	0.059231	Categorical	0.0051
Insulin (yes)	1	0.184367	Categorical	0.005044
DM (no)	0	0.343787	Categorical	0.004825
DM (yes)	1	0.343787	Categorical	0.004825
Insulin (no)	0	0.183092	Categorical	0.003987
Physcial activity (high)	2	0.094005	Categorical	0.003478
Physcial activity (miss or unknown)	3	0.066062	Categorical	0.003308
BMI	NA	0.483896	Continuous	0.003278
Antihypertensive (yes)	1	0.225066	Categorical	0.003264
Antihypertensive (no)	0	0.226333	Categorical	0.003045
Physical activity (low)	0	0.093041	Categorical	0.002977
Hypertension (no)	0	0.224957	Categorical	0.002879
Hypertension (yes)	1	0.224957	Categorical	0.002879
Alcohol (miss or unknown)	2	0.0371	Categorical	0.002562
Antihypertensive (miss or unknown)	2	0.020563	Categorical	0.002516
lowering_lipid (miss or unknown)	2	0.020563	Categorical	0.002516
Insulin (miss or unknown)	2	0.020563	Categorical	0.002516
lowering_lipid (yes)	1	0.182025	Categorical	0.002307
lowering_lipid (no)	0	0.183408	Categorical	0.002063
Physical activity (moderate)	1	0.03771	Categorical	0.001692
Race (miss or unknown)	2	0.01774	Categorical	0.001532
Alcohol (non-activie)	0	0.08493	Categorical	0.001366
Smoking (miss or unknown)	2	0.021869	Categorical	0.001267
Smoking (active)	1	0.287737	Categorical	0.001056
Diet_score (missing or unknown)	2	0.087774	Categorical	0.000939
Alcohol (active)	1	0.090756	Categorical	0.000867
Smoking (non-active)	0	0.287678	Categorical	0.000762
Diet_score (low risk)	0	0.217002	Categorical	0.000318
Diet_score (high risk)	1	0.188243	Categorical	0.000118

Table S12. Covariate Balance Assessment Before and After Inverse Probability Weighting Based on ω-6/ω-3 PUFA ratio Group Stratification (low verus high).

variable	level	Standardized Mean Difference (Unadjusted)	Type	Standardized Mean Difference (Adjusted or Weighted)
Sex (women)	1	0.147633	Categorical	0.012635
Sex (men)	0	0.147633	Categorical	0.012635
BMI	NA	0.012668	Continuous	0.007254
Hypertension (no)	0	0.162264	Categorical	0.005874
Hypertension (yes)	1	0.162264	Categorical	0.005874
Antihypertensive (yes)	1	0.176497	Categorical	0.005754
Antihypertensive (no)	0	0.176557	Categorical	0.00574
Race (White)	0	0.033539	Categorical	0.005357
Race (others)	1	0.030935	Categorical	0.005233
DM (no)	0	0.079313	Categorical	0.003898
DM (yes)	1	0.079313	Categorical	0.003898
Physical activity (low)	0	0.003141	Categorical	0.003011
lowering_lipid (yes)	1	0.312491	Categorical	0.001946
lowering_lipid (no)	0	0.311959	Categorical	0.001939
Physical activity (missing or unkown)	3	0.031449	Categorical	0.001762
Physical activity (high)	2	0.018857	Categorical	0.001754
Age	NA	0.396393	Continuous	0.001696
Race (missing or unkown)	2	0.013045	Categorical	0.001115
Physical activity (moderate)	1	0.049775	Categorical	0.001068
Diet_score (missing or unkown)	2	0.078077	Categorical	0.00094
Insulin (yes)	1	0.007258	Categorical	0.000763
Diet_score (low risk)	0	0.266403	Categorical	0.000725
Insulin (no)	0	0.005151	Categorical	0.000722
Smoking (active)	1	0.170723	Categorical	0.000412
Smoking (non-active)	0	0.16597	Categorical	0.000402
Diet_score (high risk)	1	0.246241	Categorical	0.00032
Alcohol (missing or unkown)	2	0.007558	Categorical	0.000262
Alcohol (active)	1	0.102055	Categorical	0.000255
Alcohol (non-active)	0	0.102104	Categorical	0.00021
Smoking (missing or unkown)	2	0.00824	Categorical	9.70E-06
Antihypertensive (missing or unkown)	2	0.005602	Categorical	9.37E-06
lowering_lipid (missing or unkown)	2	0.005602	Categorical	9.37E-06
Insulin (missing or unkown)	2	0.005602	Categorical	9.37E-06

Table S13. Hazard Ratios and Effective Sample Sizes for the Association Between PhenoAgeAccel, ω-6/ω-3 PUFA ratio and HF/AF Transitions After Inverse Probability Weighting

	**Effective Sample Size**		HR(95%)	P value
	Unweighted	Adjusted		
ω-6/ω-3 PUFA ratio			AF	
Low	115280	108303	Ref.	
High	75811	66002	1.26 (1.21-1.32)	<0.001
			HF	
Low	115280	108303	Ref.	
High	75811	66002	1.57 (1.48-1.67)	<0.001
			Comorbidity	
Low	115280	108303	Ref.	
High	75811	66002	1.60 (1.47-1.75)	<0.001
PhenoAgeAccel			AF	
Deceleration/Stasis	140798	129498	Ref.	
Acceleration	50293	40010	1.15 (1.10-1.19)	<0.001
			HF	
Deceleration/Stasis	140798	129498	Ref.	
Acceleration	50293	40010	1.24 (1.17-1.31)	<0.001
			Comorbidity	
Deceleration/Stasis	140798	129498	Ref.	
Acceleration	50293	40010	1.35 (1.24-1.47)	<0.001

Table S14. Multistate associations of PhenoAgeAccel and the ω-6/ω-3 PUFA ratio with AF–HF transitions after IPW.

**Multi-state model**		
**PhenoAgeAccel**		
Acceleration verus Deceleration/Stasis		
Baseline → AF	1.25 (1.20, 1.31)	<0.001
Baseline → HF	1.66 (1.54, 1.80)	<0.001
AF → Comorbidity	1.41 (1.25, 1.59)	<0.001
HF → Comorbidity	1.08 (0.87, 1.33)	0.498
**ω-6/ω-3 PUFA ratio**		
Low versus high		
Baseline → AF	1.11 (1.06, 1.16)	0.001
Baseline → HF	1.18 (1.10, 1.28)	<0.001
AF → Comorbidity	1.23 (1.09, 1.39)	<0.001
HF → Comorbidity	1.22 (0.98, 1.51)	0.069

Table S15. E-values for Key Transitions in HF/AF transitions.

**PhenoAgeAccel**			
Per 1-SD increase	HR (95% CI)	P value	E-value
Baseline → AF	1.12 (1.10, 1.15)	<0.001	1.49
Baseline → HF	1.24 (1.21, 1.26)	<0.001	1.79
AF → Comorbidity	1.12 (1.09, 1.15)	<0.001	1.49
HF → Comorbidity	1.06 (1.01, 1.12)	0.02	1.31
Acceleration verus Deceleration/Stasis			
Baseline → AF	1.20 (1.15, 1.26)	0.001	1.69
Baseline → HF	1.67 (1.55, 1.81)	<0.001	2.73
AF → Comorbidity	1.38 (1.23, 1.55)	<0.001	2.10
HF → Comorbidity	1.06 (0.86, 1.30)	0.588	-
**ω-6/ω-3 PUFA ratio**			
Per 1-SD increase			
Baseline → AF	1.04 (1.02, 1.06)	<0.001	1.24
Baseline → HF	1.07 (1.05, 1.10)	<0.001	1.34
AF → Comorbidity	1.12 (1.07, 1.18)	<0.001	1.49
HF → Comorbidity	1.10 (1.01, 1.2)	0.022	1.43
Low versus high			
Baseline → AF	1.07 (1.03, 1.12)	0.001	1.34
Baseline → HF	1.18 (1.09, 1.27)	<0.001	1.64
AF → Comorbidity	1.23 (1.09, 1.37)	<0.001	1.76
HF → Comorbidity	1.14 (0.93, 1.40)	0.199	-

Figure 1. Multistate transition framework for AF, HF, and AF–HF comorbidity.
Arrows denote the four modeled transitions: (1) baseline→AF; (2) baseline→HF; (3) AF→AF–HF comorbidity; (4) HF→AF–HF comorbidity. Participants with AF and HF diagnosed on the same day (n=757) were excluded because temporal ordering could not be determined. Comorbidity was defined as the state in which a participant had experienced both atrial fibrillation and heart failure during follow-up, irrespective of sequence.

Figure S1

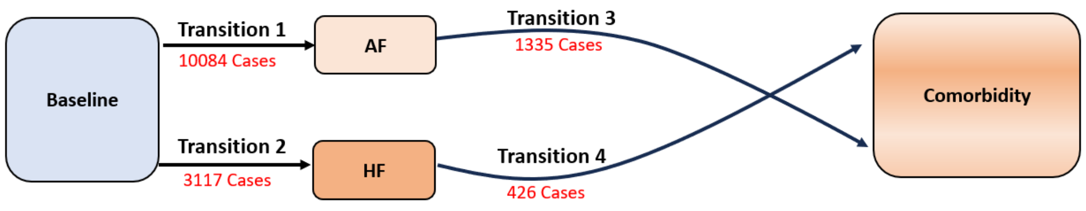

Figure S2. Directed acyclic graph of the link between PhenoAgeAccel, ω-6/ω-3 PUFA ratio and the incidence of HF/AF.

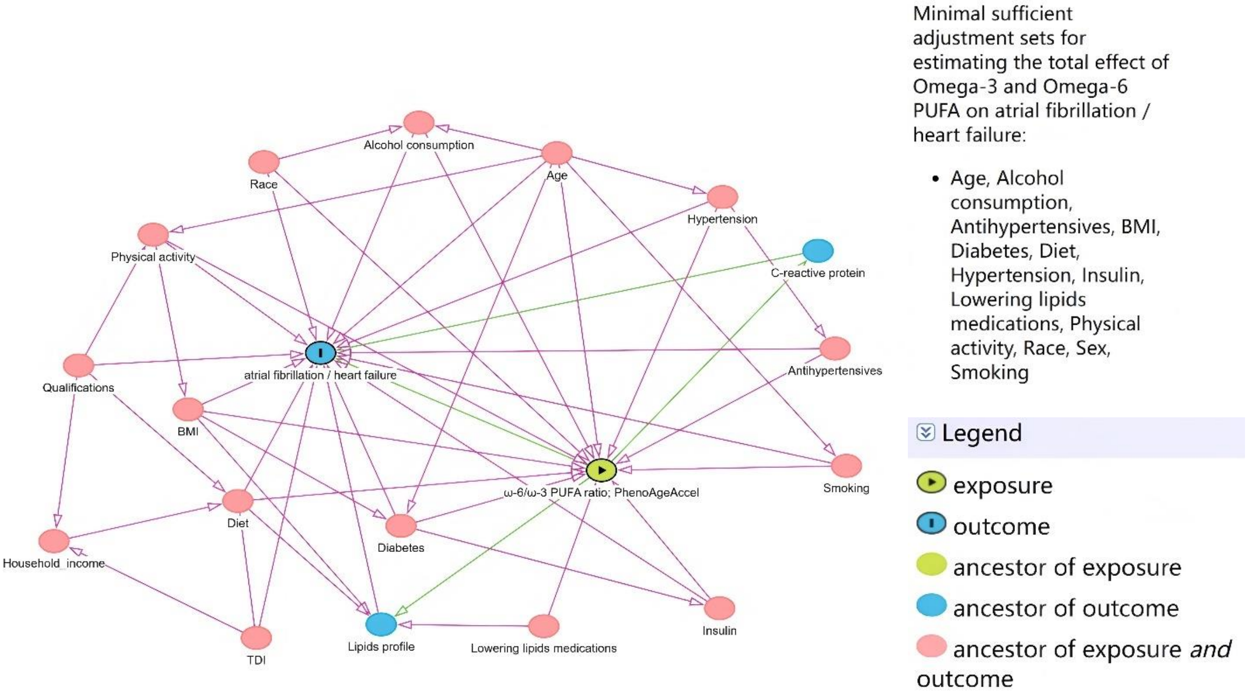

Abbreviations: BMI, body mass index; PhenoAgeAccel, phenotypic age acceleration; AF, atrial fibrillation; HF, heart failure; ω-6/ω-3 PUFA ratio, ω-6 : ω-3 polyunsaturated-fatty-acid ratio.

In the DAG diagram, green ovals represent the exposure, and blue ovals represent the outcome. Light green and light blue nodes indicate variables that are ancestors of the exposure and outcome, respectively. Pink nodes are shared ancestors of both exposure and outcome. Causal paths are shown as green arrows, and potential biasing paths are marked in purple.

Notes:

The graph was created with the help of DAGitty.net (www.dagitty.net). Minimally sufficient adjustment set: age, body mass index, sex, race, physical activity, hypertension, diabetes, cumulative dietary-risk score, smoking status, alcohol consumption, use of antihypertensives, lipid-lowering agents, and insulin.

Figure S3. Schoenfeld Residuals for the Association Between the ω-6/ω-3 PUFA Ratio and Incident Heart Failure.

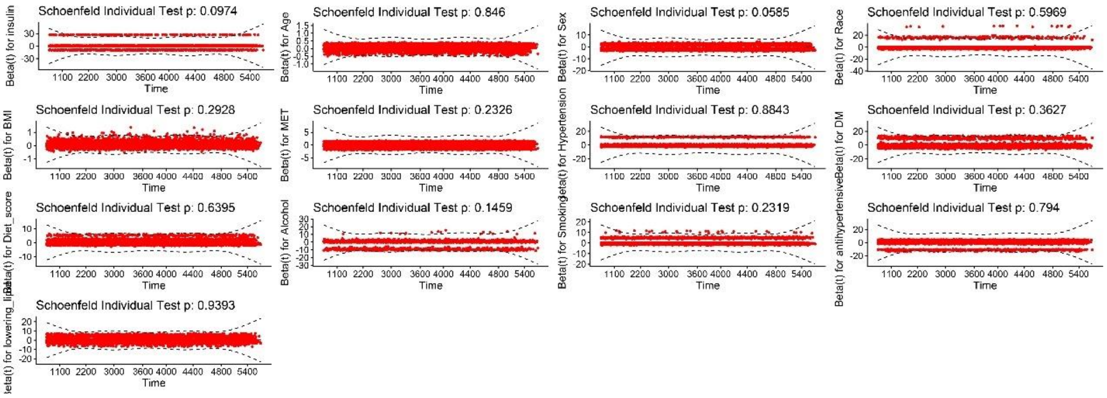

Figure S4. Schoenfeld Residuals for the Association Between the ω-6/ω-3 PUFA Ratio and Incident atrial fibrillation.

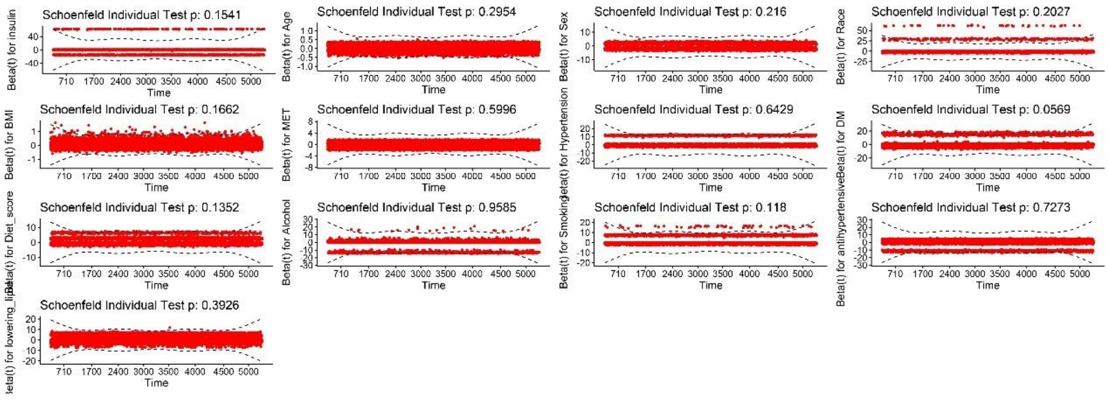

Figure S5. Schoenfeld Residuals for the Association Between the ω-6/ω-3 PUFA Ratio and Incident comorbidity.

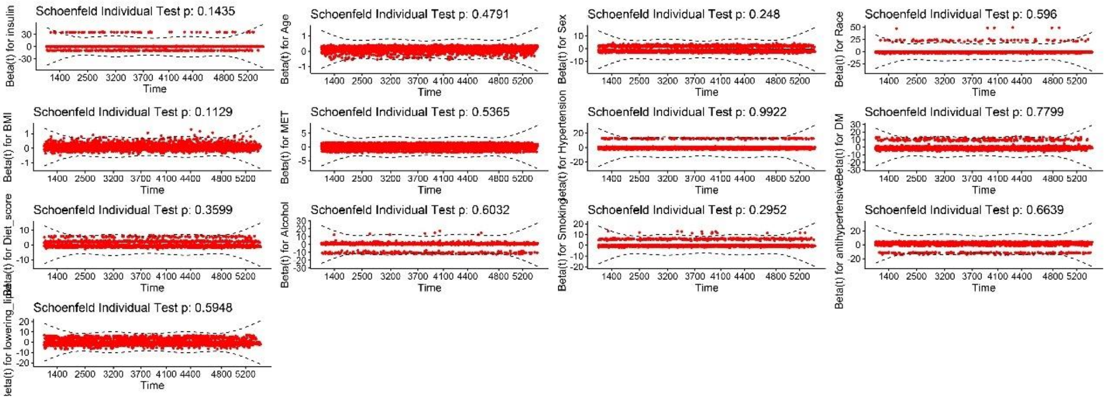

Figure S6. Schoenfeld Residuals for the Association Between PhenoAgeAccel and Incident Heart Failure.

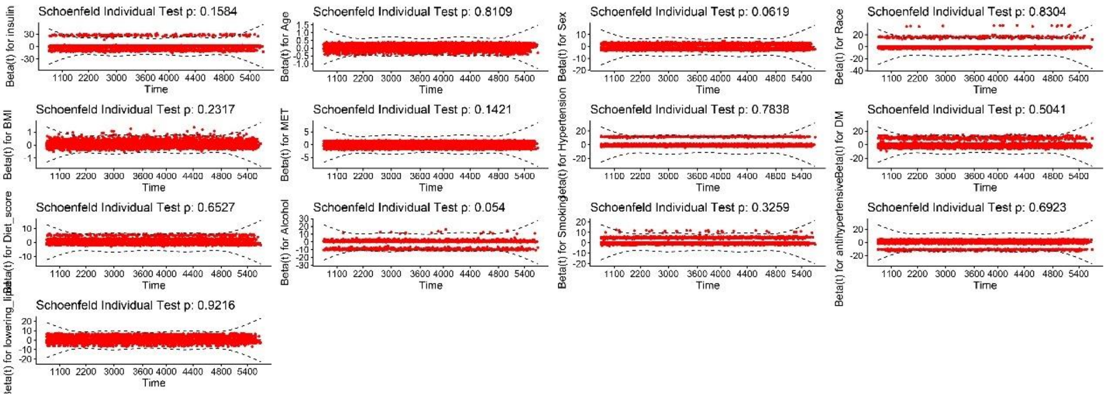

Figure S7. Schoenfeld Residuals for the Association Between PhenoAgeAccel and Incident atrial fibrillation.

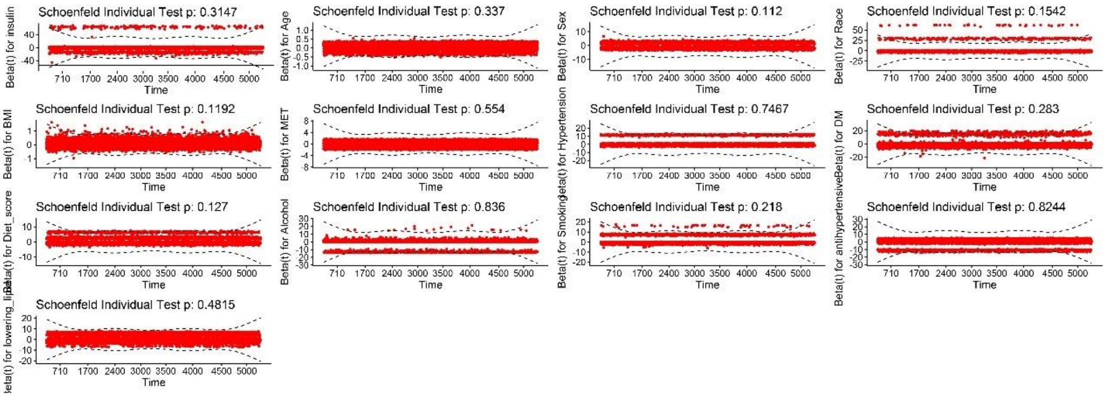

Figure S8. Schoenfeld Residuals for the Association Between PhenoAgeAccel and Incident comorbidity.

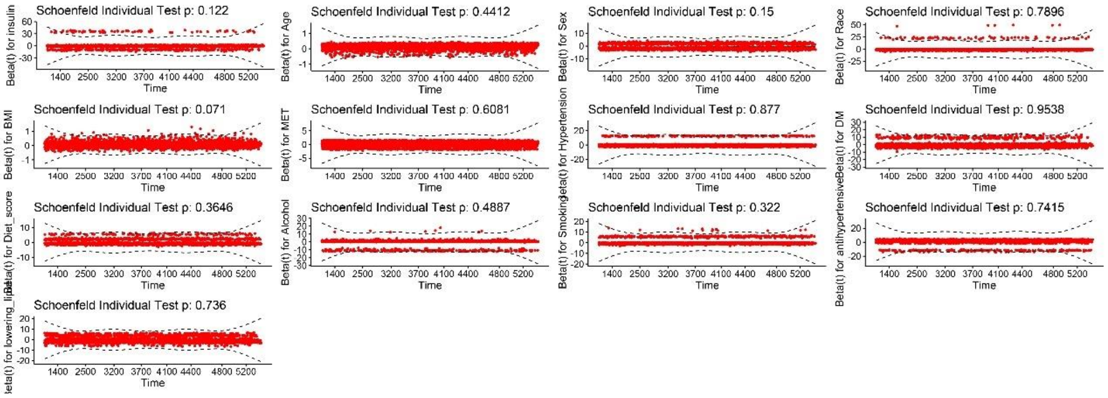

Figure S9. Penalized spline analyses (df = 3) of associations between ω-6/ω-3 PUFA ratio, PhenoAgeAccel and transitions of atrial fibrillation, heart failure, and comorbidity. Models were adjusted for age, body mass index, sex, race, physical activity, hypertension, diabetes, diet score, smoking status, alcohol consumption, antihypertensive use, lipid-lowering medication use, and insulin use.

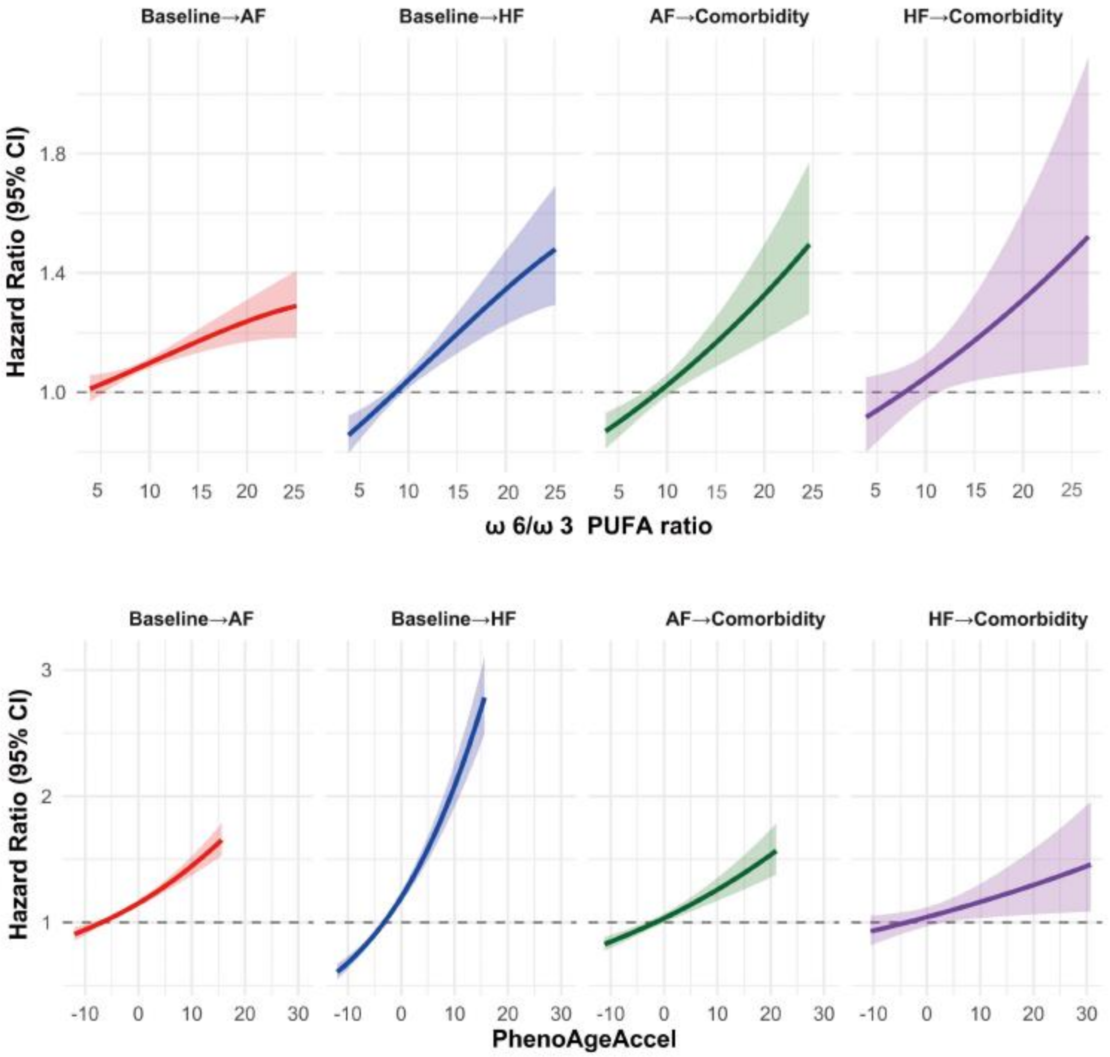

Figure S10. Restricted cubic spline exposure–response curves for PhenoAgeAccel and the ω-6/ω-3 PUFA ratio across AF–HF transition hazards.

PhenoAgeAccel, phenotypic age acceleration; AF, atrial fibrillation; HF, heart failure; ω-6/ω-3 PUFA ratio, ω-6 : ω-3 polyunsaturated-fatty-acid ratio.

All models were adjusted for age, body mass index, sex, race, physical activity, hypertension, diabetes, cumulative dietary-risk score, smoking status, alcohol consumption, use of antihypertensives, lipid-lowering agents, and insulin.

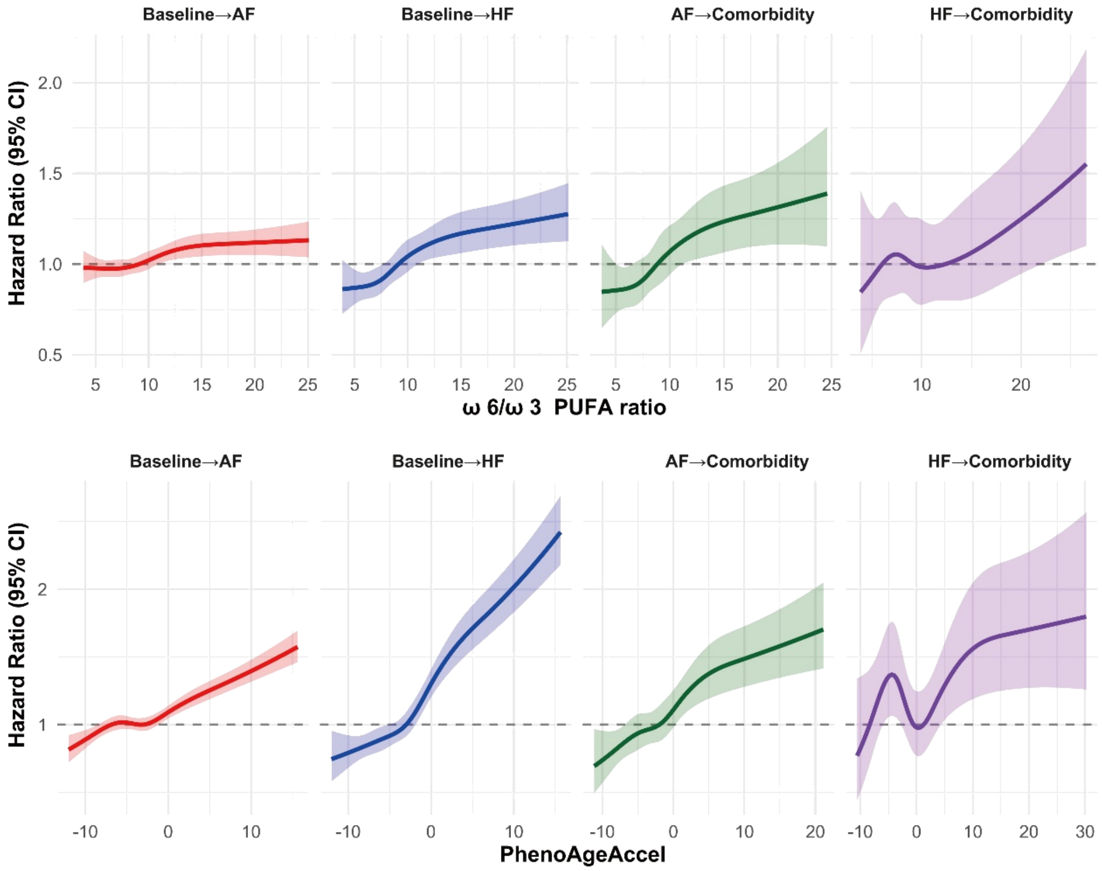

Figure S11. Mediation analysis of lipid profile and C-reactive protein linking PhenoAgeAccel to AF–HF transition risk.

PhenoAgeAccel, phenotypic age acceleration; AF, atrial fibrillation; HF, heart failure; ω-6/ω-3 PUFA ratio, ω-6 : ω-3 polyunsaturated-fatty-acid ratio.

Mediation models were adjusted for the following covariates: age, body mass index, sex, race, physical activity, hypertension, diabetes, cumulative dietary risk score, smoking status, alcohol consumption, and use of antihypertensives, lipid-lowering agents, and insulin.

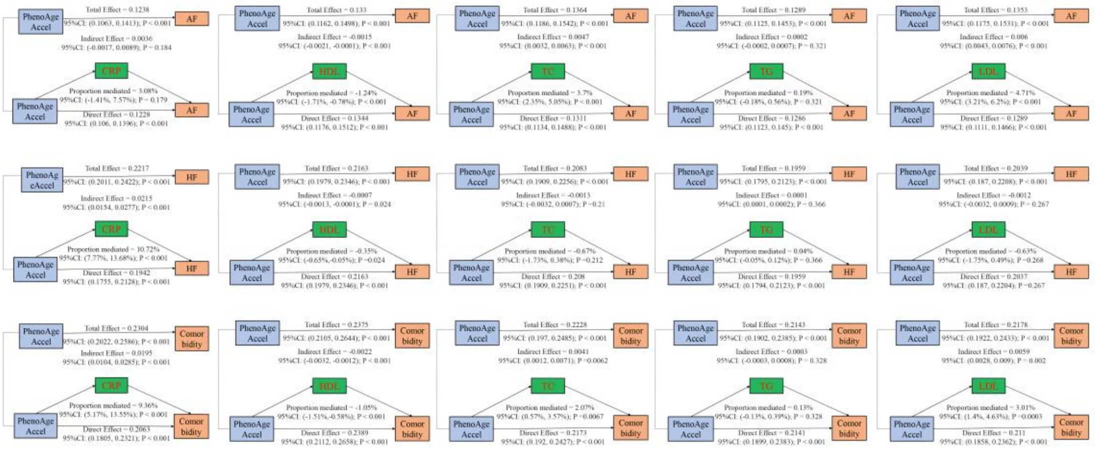

Figure S12. Mediation analysis of lipid profile and C-reactive protein linking the ω-6/ω-3 PUFA ratio to AF–HF transition risk.

PhenoAgeAccel, phenotypic age acceleration; AF, atrial fibrillation; HF, heart failure; ω-6/ω-3 PUFA ratio, ω-6 : ω-3 polyunsaturated-fatty-acid ratio.

Mediation models were adjusted for the following covariates: age, body mass index, sex, race, physical activity, hypertension, diabetes, cumulative dietary risk score, smoking status, alcohol consumption, and use of antihypertensives, lipid-lowering agents, and insulin.

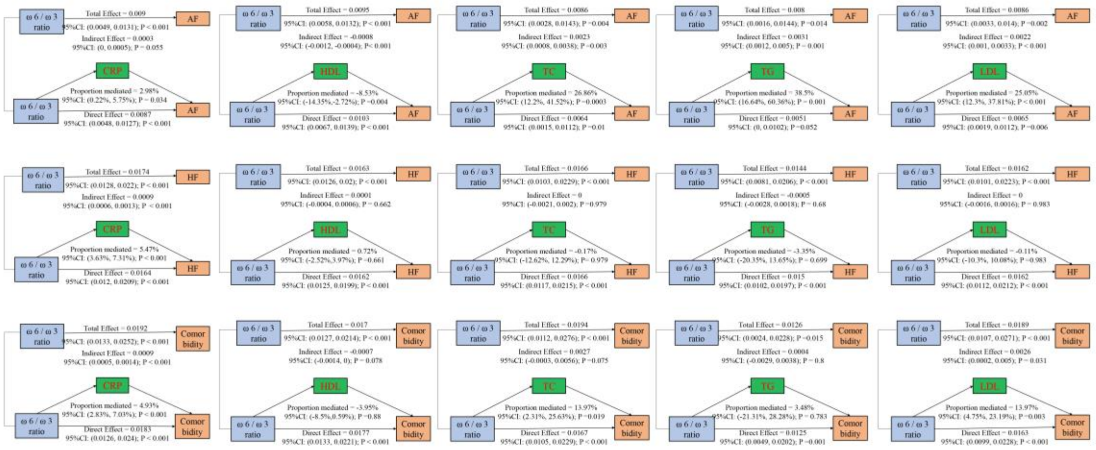

Figure S13 Subgroup Analyses of Phenotypic Age Acceleration and the ω-6/ω-3 PUFA Ratio Across Transition Pathways to AF–HF Comorbidity.

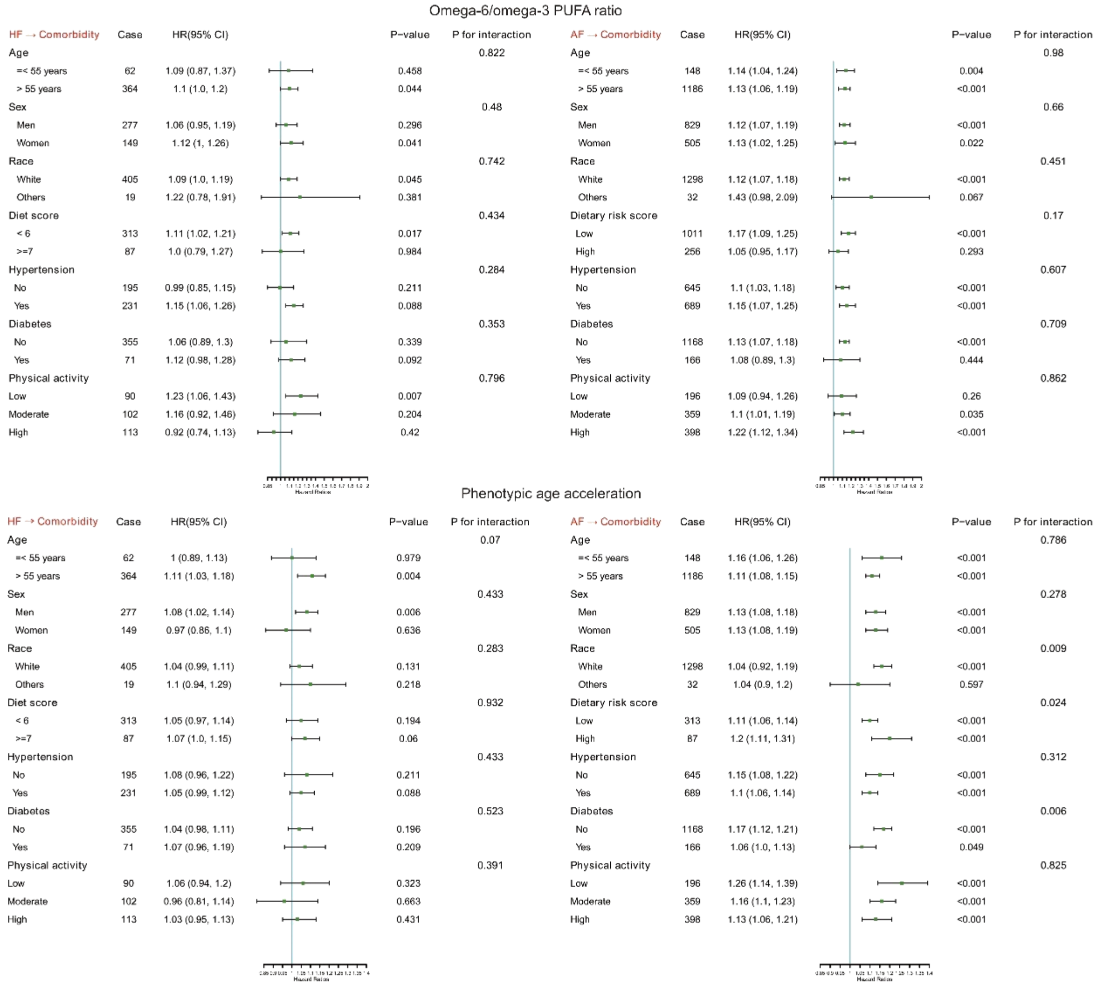

Supplement: Supplementary file 1 [file mmc1.pdf]
